# Arterial tracing‐based technique for rapid purification of pulmonary arterial smooth muscle cells from rat lungs

**DOI:** 10.14814/phy2.71098

**Published:** 2026-09-07

**Authors:** Lucynda Pham, Lauren Pavelich, Natascha Sommer, Lawrence I. Grossman, Maik Hüttemann, Tasnim Arroum

**Affiliations:** ^1^ Center for Molecular Medicine and Genetics Wayne State University Detroit Michigan USA; ^2^ Excellence Cluster Cardiopulmonary System University of Giessen and Marburg Lung Center (UGMLC), Member of the German Center for Lung Research (DZL), Justus‐Liebig‐University Giessen Germany; ^3^ Department of Biochemistry, Microbiology, and Immunology Wayne State University Detroit Michigan USA

**Keywords:** agar‐food dye delineation, COX4i2, hypoxia, mitochondria, primary cell isolation, pulmonary smooth muscle cell

## Abstract

Primary pulmonary arterial smooth muscle cells (PASMCs) of pre‐capillary vessels are used to study the molecular mechanism of hypoxic pulmonary vasoconstriction (HPV). Cytochrome c oxidase subunit IV isoform 2 (COX4i2) is highly expressed in these PASMCs and an essential player in HPV, as its knockout abolishes the response. However, the exact molecular mechanism is not fully understood; therefore, fast and reliable methods to isolate PASMC from small pulmonary vessels that retain their phenotype during culture are essential. In the current study, we aimed to develop a fast and user‐friendly method to isolate and culture homogeneous PASMCs from rat lungs. PASMCs were isolated utilizing agar mixed with a food‐safe blue dye to delineate pulmonary arteries (PAs) within the lung, facilitating the tissue dissection process. Cox4i2 expression was determined by qPCR and mass spectrometry. Highly enriched populations of PASMCs expressing the classic smooth muscle cell markers α‐SMA (alpha‐smooth muscle actin) and SM‐MHC (smooth muscle myosin heavy chain) were obtained for rat lungs. The cells were cultured up to passage 4, remained homogeneous, and maintained their identity. Additionally, the cells expressed Cox4i2 under normoxia and demonstrated an increase in Cox4i2 expression after exposure to chronic hypoxia, a known PASMC response. PASMCs were easily and rapidly obtained from rat lungs and provide a cell culture model to study mechanisms of HPV in which a key player, COX4i2, is retained during the cell culture process.

## 1 INTRODUCTION

Under conditions of hypoxia, systemic arterial vessels dilate whereas the pulmonary arterial vessels constrict. Hypoxic pulmonary vasoconstriction (HPV), also known as the von Euler‐Liljestrand mechanism, is a physiological response to alveolar hypoxia that reduces blood flow to poorly ventilated areas and shunts it to well‐ventilated areas to optimize gas exchange. Thus, when oxygen partial pressure drops, oxygen‐sensing cells signal the arteries to constrict, diverting blood to better‐oxygenated areas of the lungs, maintaining ventilation to perfusion matching (V/Q ratio) to promote optimal arterial blood oxygenation (Dunham‐Snary et al., 2017; Sommer et al., 2016). Although the HPV response was recognized decades ago, the molecular pathways mediating this response are still not fully understood. The oxygen‐sensing machinery is believed to be mediated by the pulmonary arterial smooth muscle cells (PASMCs) of the precapillary vessels, with mitochondria playing a central role (Pak et al., 2022; Waypa et al., 2001), although evidence for a role of the endothelium has also been provided (Grimmer & Kuebler, 2017; Wang et al., 2012). The signal transduced from the initial sensor, which depends on the electron transport chain complex cytochrome *c* oxidase (COX) containing the oxygen‐sensing COX subunit 4 isoform 2 (COX4i2) (Sommer et al., 2017), results in a downstream increased release of mitochondrial reactive oxygen species (ROS), leading to the inhibition of voltage‐dependent potassium channels, depolarization of the plasma membrane, activation of voltage‐gated calcium (Ca^2+^) channels, and an increase in cytosolic calcium, ultimately leading to vasoconstriction.

Impairment of this response could result in hypoxemia, and a chronic state of alveolar hypoxia can lead to sustained vasoconstriction and hypoxia‐induced vascular remodeling, both established contributors to pulmonary hypertension (Pak et al., 2007). Chronic hypoxia occurs at high altitude or during certain respiratory diseases, such as sleep apnea. Pulmonary vasoconstriction and vascular remodeling are known to predominantly affect the distal pulmonary arteries (PAs) with diameters below approximately 50–100 μm (Crosswhite & Sun, 2014; Vanderpool et al., 2011). Thus, isolation and culture of PASMCs from the distal arteries provide researchers with a tool to elucidate the molecular mechanisms of HPV in response to hypoxia in a cell culture model, with the advantage of performing assays such as ROS production and Ca^2+^ release measurements, which are difficult to carry out in vivo.

### 1.1 Existing methods for PASMC isolation

Methods to isolate PASMCs involve the use of large animals such as bovine, ovine, and porcine, as the lungs are large and dissection of the medial layer is easily achieved (Lee et al., 1991; Suzuki et al., 2003; Yang et al., 2012). Limitations of these approaches are the difficulty in genetically manipulating these animals, in addition to the high costs. Another method includes the explant/tissue outgrowth method where a small piece of the dissected artery is placed directly into culture and the PASMCs grow out (McMurray et al., 1991). However, this method has a limitation as there is less control over the cell types cultured. Methods to specifically isolate pre‐capillary PASMCs with a focus on smaller animals such as mice or rats exist. One such method is manual dissection of rat lungs followed by enzymatic digestion (Peng et al., 2010, 2017; Rothman et al., 1992; Zeng et al., 2015). This method does not always allow easy identification and correct isolation of the pre‐capillary PASMCs, which is particularly difficult in smaller animal models such as rodents. To specifically enrich for pre‐capillary distal PASMCs, the iron particle and agarose perfusion method was established: lungs are perfused with iron oxide particles embedded in agarose, small intra‐acinar arteries are magnetically isolated, and the tissue is enzymatically digested. The iron‐particle infusion method was developed and further optimized by different labs (Johnson et al., 1994; Marshall et al., 1996; Waypa et al., 2001). This method was even shown to work in neonatal mice (Lee et al., 2013).

### 1.2 Novel method to isolate PASMCs by intra‐arterial tracing

**FIGURE 1:**
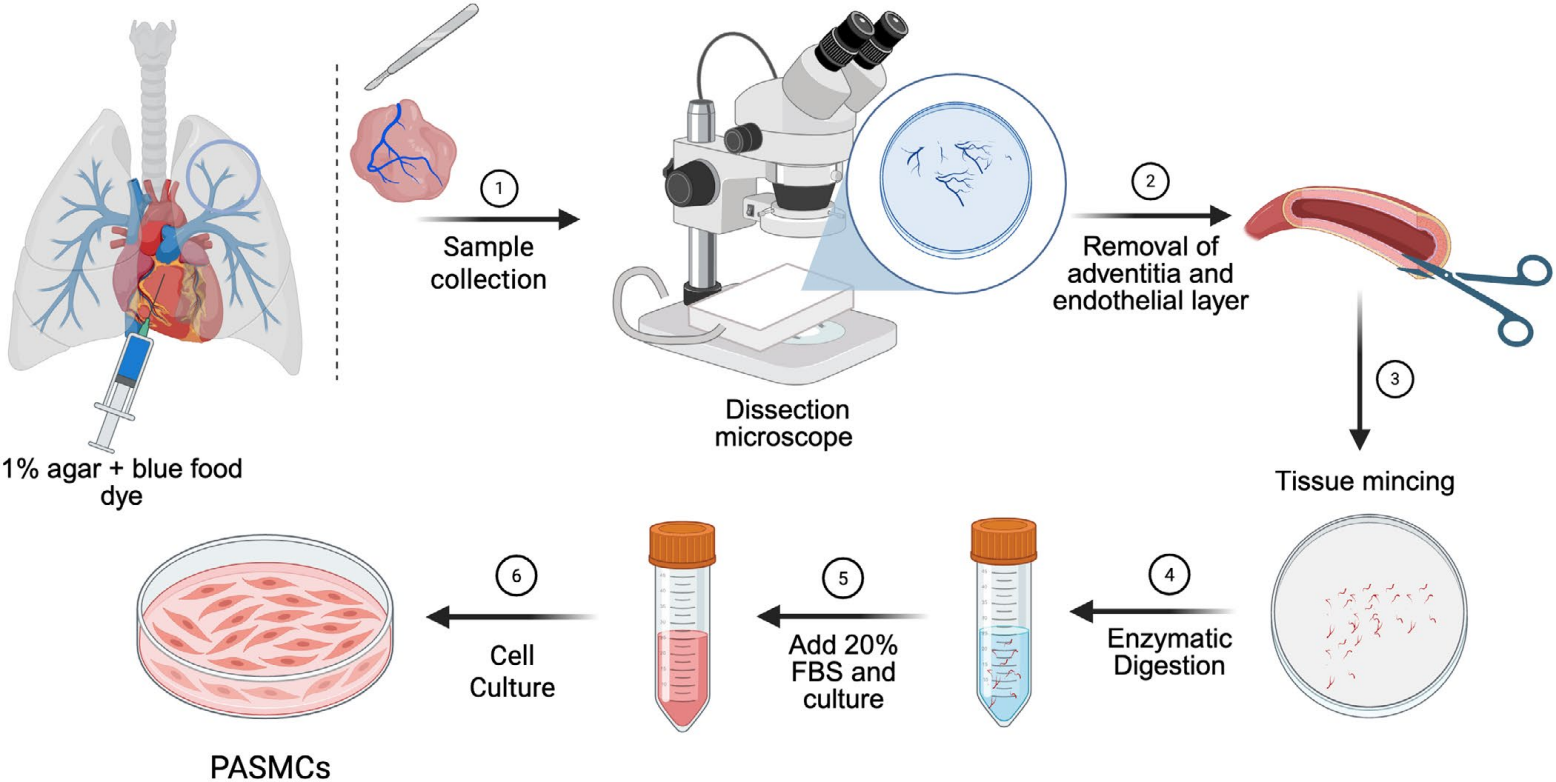
Schematic workflow of PASMC isolation from lungs utilizing injection of agar supplemented with blue food dye to delineate and isolate distal pulmonary arteries (PAs).

Manual dissection approaches tend to sample larger extrapulmonary or proximal intrapulmonary branches and therefore may not accurately represent pre‐capillary PAs. These challenges arise largely from the difficulty of reliably identifying small pre‐capillary PAs in intact lungs, particularly for less experienced investigators. Here, we present a simple, cost‐effective, and alternative technique to typical manual dissection methods to isolate PAs and obtain highly enriched PASMCs from rat lungs. By selectively filling and staining the arterial tree, the agar–blue dye method overcomes limitations of manual dissection and enables distal PA identification between operators. Our methodology utilizes a food‐grade blue dye mixed with agar for intra‐arterial tracing combined with anatomical dissection and collagenase‐based enzymatic digestion to achieve clear vessel delineation (Figure 1). Finally, we characterized cell morphology, markers of contractile smooth muscle cells (SMCs), and chronic hypoxic response, and found that the blue dye dissection method works well for rat PASMCs derived from lungs as they retain expression of markers associated with HPV.

## 2 MATERIALS AND METHODS

### 2.1 Rat PASMC isolation

#### 2.1.1 Delineation and isolation of distal pulmonary arteries

**FIGURE 2:**
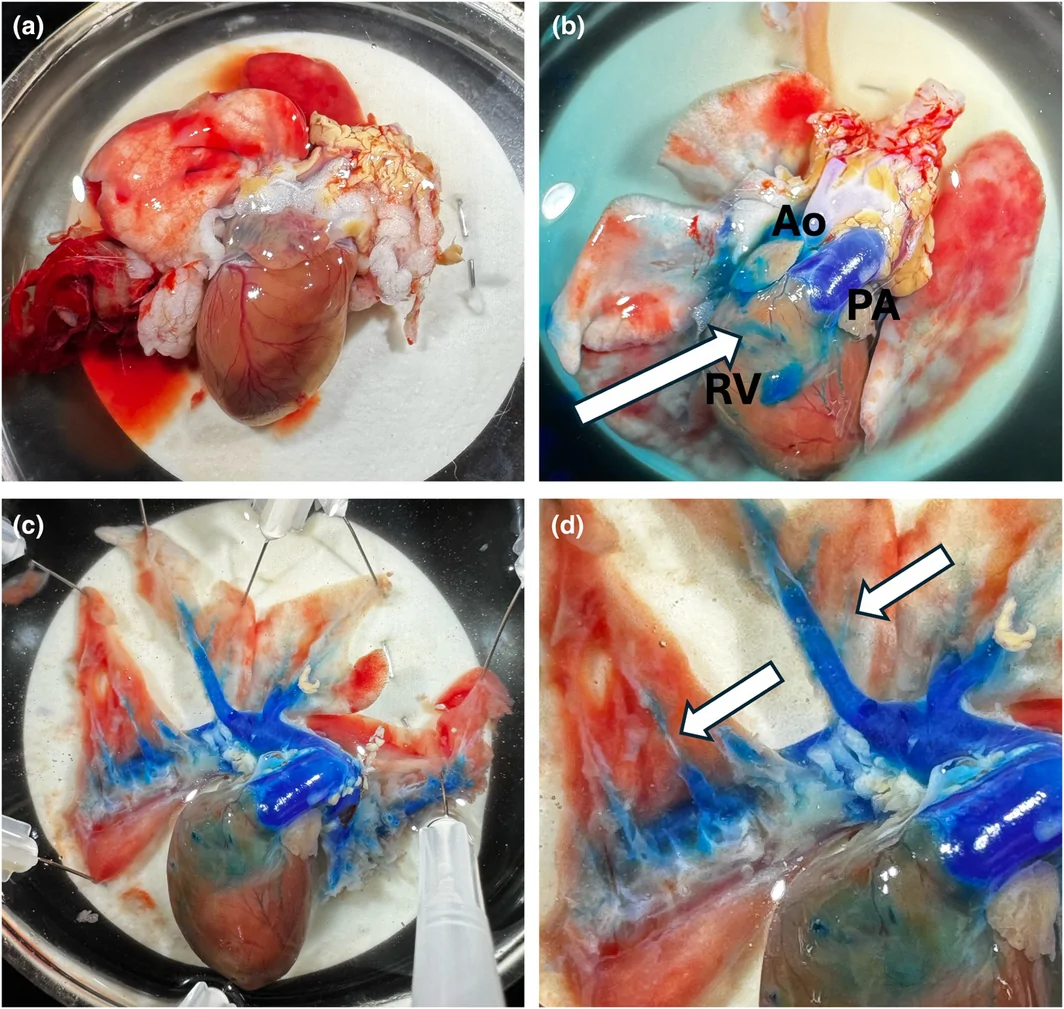
Pulmonary artery tracing and dissection from rat lungs. (a) Heart and lung were removed *en bloc* in ice cold physiological salt solution (PSS). (b) Lungs were perfused with cold PBS by injection in the right ventricle (RV) followed by injection with 1% agar supplemented with food‐safe blue dye to delineate the pulmonary arteries (PA). (c) Excess tissue was removed to reveal the PAs. (d) The distal PAs >3rd generation were dissected out. Ao, aorta.

Male Sprague Dawley rat (age: 6 months) plucks (hearts still connected with the lung) were obtained as discarded tissues under Wayne State University's IACUC protocol 22‐04‐4589 immediately after animals were euthanized with an overdose injection of sodium pentobarbital (200 mg/kg, i.p.). A total of 3 rats were used for this study. Pluck dissections of rat hearts and lungs, removed *en bloc*, was carried out immediately following euthanasia by accessing the thoracic cavity, freeing the trachea and esophagus, and cutting them near the top of the neck area, followed by cutting the connective tissue around the heart and diaphragm (Figure 2a). The organs were placed in cold (4°C) physiological salt solution (PSS) containing 130 mM NaCl, 5 mM KCl, 1.2 mM MgCl_2_, 10 mM HEPES, and 10 mM glucose. The lungs were perfused with 10 mL of ice‐cold sterile phosphate buffered saline (PBS) by injection via a 10 mL syringe into the right ventricle (RV), followed by injection of 3 mL of 1% agar (Fisher Scientific, Waltham, MA, #BP1425‐500) *supplemented with a food dye as follows*: The agar was brought to a boil and then cooled to 50°C, supplemented with 2–3 drops of food‐safe blue dye (Chef Master, Royal blue Liqua‐Gel liquid food coloring, #5415; this product contains food dyes FD&C BLUE 1 (E133) and FD&C RED 3 (E127)), and the solution was allowed to cool to 37°C before injection into the RV as above to prevent heat damage to the cells. Injections were performed using 5 mL syringes and 25G needles. After the blue agar solution was injected, it solidified into a soft gel, staining and tracing the PAs (Figure 2b). The addition of agar provided increased tissue stability, facilitating easier isolation. The blue coloration enabled clear identification of the arterial structures and prevented buffer and dye perfusion into other non‐target structures. Due to the viscosity of the agar solution, the solidified agar clogged the capillaries, allowing easy identification of distal PAs. It is important to ensure that the agar solution remains liquid and does not coagulate inside the syringe or the needle; therefore, this step should be carried out quickly.

#### 2.1.2 Medial layer manual dissection

The various lobes of the lung were pinned down using syringe needles for better visualization. Excess lung tissue and veins, including the aorta, were removed to better delineate and visualize the PAs using micro‐forceps (Figure 2c). The distal PAs (3rd or greater generations) were collected as annotated by arrows (Figure 2d). Under a dissection microscope, the adventitial layer was peeled away with fine micro‐forceps. The arteries were subsequently cut longitudinally to expose the luminal surface starting at larger diameter and following down the arterial branches using micro‐scissors and continuing into the 3rd and 4th generation until reaching arteries where the dye was no longer visible. Endothelial cell denudation was performed by mechanical stripping and removed by gently positioning the opened vessel between the tips of fine forceps. A second pair of forceps was used to stabilize the vessel while the first pair of forceps was gently pulled along the length of the artery to mechanically detach and remove the endothelial sheet. Alternatively, the use of a cotton swab to gently rub and remove the endothelial layer. The remaining tissue was cut into small pieces using surgical scissors. From this point onward, all subsequent procedures were conducted under sterile conditions in a cell culture hood to minimize contamination.

#### 2.1.3 Enzymatic digestion and culture

**FIGURE 3:**
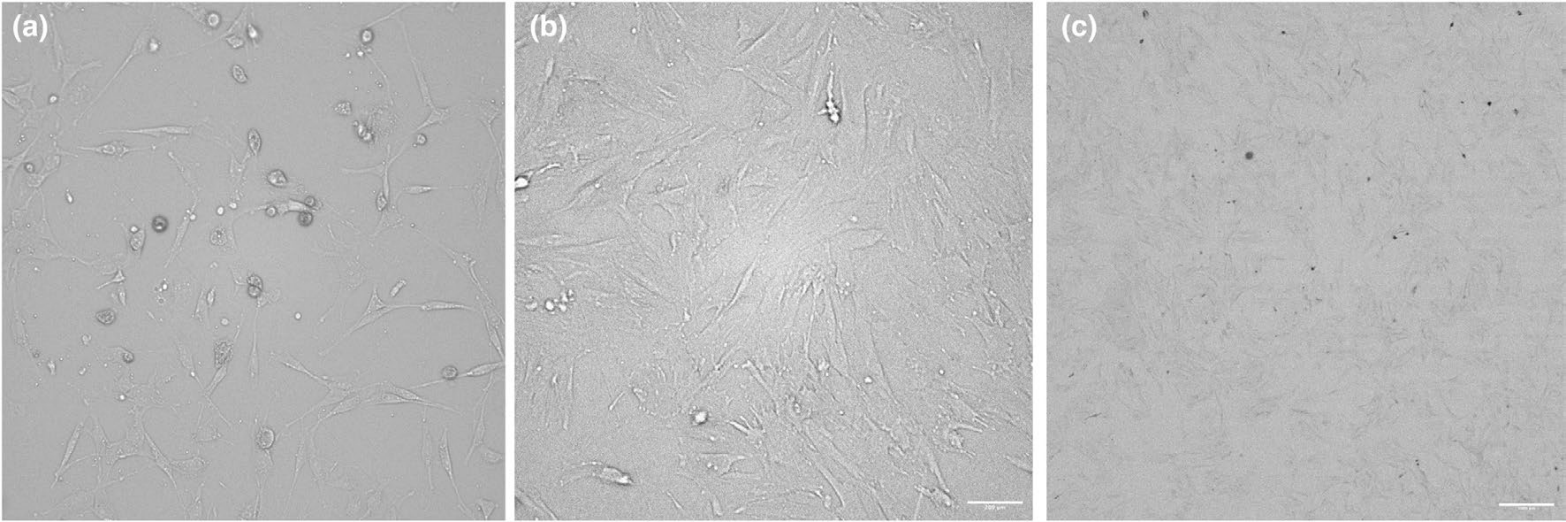
Morphological assessment of primary PASMCs. Representative brightfield images of isolated cells from rat lungs, (a) 3 days post isolation and (b) 7 days post‐isolation at 20X magnification; scale bar 200 μm. (c) Confluency was reached after about 7 days (4X magnification; scale bar 1000 μm). A total of 3 independent pluck preparations were performed and confirmed. Data presented represents a single isolation.

The tissue (20–50 mg) was then incubated with 10 mL Dulbecco's modified Eagle's medium (DMEM) (4.5 g/L glucose) (Gibco, Grand Island, NY, USA, #11965–092) without FBS supplemented with Collagenase Type A (Roche, Basel, Switzerland, #10103578001) and Type II (Stemcell Technologies, Vancouver, Canada, #07418) (10 μg/mL each) for 1 h at 37°C in a 15 mL conical tube placed in a water bath. The tissue was then spun down, and the media was removed by gently pipetting the liquid. To the tissue pieces, 10 mL DMEM + 20% FBS + 1% penicillin/streptomycin (P/S) were added, and the tissue was resuspended with a P1000 micropipette and triturated to free the cells from any remaining tissue pieces. The cells were placed into cell culture dishes and maintained at 37°C and 5% CO_2_. Cells began to attach, and colonies were visible around 3–5 days and displayed the spindle‐shaped morphology characteristic of SMCs (Figure 3a). Half media changes were done every 2–3 days. Once all cells attached and became confluent within about 1 week, to continue culturing the cells they were split once per week, with each split defining a new passage.

### 2.2 Immunofluorescence staining

Cells were seeded onto glass slides and fixed with ice‐cold methanol: acetone (1:1) for 5 min. Slides were washed with cold PBS 3 X 5 min and then permeabilized with 0.1% Triton‐X 100 in PBS for 5 min, followed by 3 washes with PBS. Slides were blocked with 10% goat serum (Abcam, Cambridge, UK, #ab7481) + 1% BSA (Millipore, Burlington, MA, USA, #81‐053‐3) in PBS for 1 h at room temperature. Primary antibodies used with dilutions include α‐SMA (1:500, ProteinTech, Rosemont, IL, USA, #80008‐1‐RR) at 1:500, SDHA (1:500, Abcam, Cambridge, UK, #ab14715), and SM‐MHC (1:500, ProteinTech, #21404‐1‐AP). Primary antibodies were incubated overnight at 4°C. Slides were then washed 3 X 5 min. Secondary antibodies and dilutions, including goat anti‐rabbit IgG H&L (Alexa Fluor 488) (1:1000, Abcam, #ab150077) and goat anti‐mouse‐IgG H&L (Alexa Fluor 594) (1:500, Abcam, #ab150116) at 1:500 dilutions, were incubated for 1 h at room temperature, followed by 3 washing steps with PBS for 5 min each. Nuclei were stained with ProLong Gold Antifade with DAPI (Invitrogen, Carlsbad, CA, USA, #P36941), and coverslips were mounted. Images were obtained using the BioTek Cytation C10 (BioTek Instruments Inc., Winooski, VT, USA).

### 2.3 Chronic hypoxia exposure

Cells were cultured and maintained in DMEM with 20% FBS and 1% P/S until ready to seed. Cells cultured in the differentiation media stop proliferating; therefore, PASMCs were first seeded into the appropriate experimental culture vessel at the required density. At passage 4 (approximately 20 days in culture), cells were adapted for 3 days to low‐glucose DMEM (1 g/L glucose; Gibco, Grand Island, NY, USA, #11885084) supplemented with heparin (30 μg/mL) (Sigma Aldrich, St. Louis, MO, USA, #H3149‐10KU), TGFβ1 (10 ng/mL; RND Systems, Minneapolis, MN, USA, #7754‐BH), and 1% FBS and sterile filtered. The differentiation medium was prepared fresh and stored at 4°C for no longer than 2 weeks. TGFβ1 (10 μg/mL) stock solutions were prepared, aliquoted, and stored at −80°C until use. Cells were then placed in a hypoxia chamber at 37°C with 1% O_2_ and 5% CO_2_ controlled by ProOx 110 Compact O_2_ Controller (BioSpherix; Redfield, NY, USA, RRID: SCR_021129) and ProCO_2_ 120 Compact CO_2_ Controller (BioSpherix, RRID: SCR_021127) for 48 h.

### 2.4 RT‐qPCR

RNA was isolated using the TRIzol/chloroform method as described (Rio et al., 2010). Briefly, cells were vortexed in TRIzol (Invitrogen, #15596026) followed by the addition of chloroform and vortexed again. The suspension was centrifuged at max speed in a table‐top centrifuge for 15 min. The aqueous layer was collected, precipitated with isopropanol, and centrifuged again. The RNA pellet was allowed to dry, washed with 75% ethanol, centrifuged, and dried again. The pellet was reconstituted in water and RNA concentration was quantified using a NanoDrop One spectrophotometer (ThermoFisher, Waltham, MA, USA, ND‐ONE‐W). Total cDNA was synthesized from isolated RNA using the iScript cDNA synthesis kit (Biorad, Hercules, CA, USA, #1708891) according to the manufacturer's protocol. RT‐qPCR was performed using the PowerUp SYBR Green master mix (Applied Biosystems, Waltham, MA, USA, #A25776). RT‐qPCR using 96‐well plates (Applied Biosystems, #4346906) was run using the 7500 Fast Real‐Time PCR System (Applied Biosystems). PCR primers and sequences are listed in Table 1.

**TABLE 1:** Primer sequences for rat qPCR.

GENE	*Cox4i2*	*Cox4i1*	*Gapdh*
Forward Sequence	5′‐CCCTATGTTGACTGCTATGCTC‐3′	5′‐ATGTTGATCGGCGTGACTAC‐3′	5′‐ACCTGCCAAGTATGATGA‐3′
Reverse Sequence	5′‐AGCCCATTACTGTCTTCCATTC‐3′	5′‐CACCACTGTCTTCCACTCATT‐3′	5′‐GGAGTTGCTGTTGAAGTC‐3′

### 2.5 Mitochondria isolation from cells

Mitochondria were isolated from rat PASMCs exposed to normoxia (NOX) or hypoxia (HOX) for 48 h at 1% oxygen according to previously published protocols (Pham et al., 2025). Briefly, cells were resuspended in hypotonic buffer (10 mM NaCl, 1.5 mM MgCl_2_, 10 mM Tris–HCl (pH 7.5)) and mechanically disrupted using a Teflon homogenizer (80–120 strokes, 1500 rpm). Mitochondrial stabilization buffer (MSB; 210 mM mannitol, 70 mM sucrose, 5 mM Tris–HCl (pH 7.5), 1 mM EDTA (pH 7.5)) was added and centrifuged at 800 *× g* for 5 min at 4°C. The supernatant was transferred to a new tube and centrifuged at 10,000 *× g* for 10 min to obtain the mitochondrial pellet. The pellets were split into 50 μg aliquots.

### 2.6 Peptide preparation and mass spectrometry analysis

Peptides were generated from isolated rat mitochondria using S‐Trap™ mini columns (ProtiFi, Farmingdale, NY, USA, #C02‐mini‐80) according to the manufacturer's instructions. Following the lysis step, total protein concentration was quantified using DC™ Protein Assay (Bio‐Rad, Hercules, CA, USA, #5000111). A total of 100 μg protein was digested overnight at 37°C in a water bath using 10 μg trypsin protease (Thermo Fisher Scientific, Waltham, MA, USA, #90058). The concentration of eluted peptides was determined using the Pierce™ Quantitative Peptide Assay and Standards assay (Thermo Fisher Scientific, Waltham, MA, USA, #23275). Protein abundance was measured by label‐free proteomic analysis at Wayne State University's Proteomics Core Facility (Detroit, MI, USA). Samples were analyzed using a Thermo Scientific Orbitrap Astral Zoom mass spectrometer coupled to an Evosep Eno LC system. Peptides were loaded onto Evosep C18 tips and separated on an IonOpticks Aurora Elite XT column (75 μm × 15 cm) using the 40 samples‐per‐day (SPD) Whisper Zoom method. Solvent A consisted of 0.1% formic acid (FA) in water, and solvent B consisted of 99.9% acetonitrile and 0.1% FA. Mass spectrometric analysis was performed in data‐independent acquisition (DIA) mode using a nano‐electrospray ionization source. MS1 spectra were acquired using the Orbitrap at a resolution of 240,000 over an *m*/*z* range of 380–980, with an RF lens setting of 40%. MS2 spectra were acquired in the Astral analyzer using HCD with a collision energy of 25%, 300 sequential isolation windows of 2 *m*/*z*, a precursor scan range of 380–980 *m*/*z*, an AGC target of 500%, and a maximum injection time of 2.5 ms. Raw data were processed using Spectronaut 21.0, searching against the *Rattus norvegicus* (UP000002494), Uniprot FASTA database (downloaded March 2026, 9894 entries; UniProt (https://www.uniprot.org)). Search parameters allowed up to 2 missed cleavages from trypsin digestion. Carbamidomethylation of cysteine was specified as a fixed modification, while oxidation of methionine, deamidation of asparagine and glutamine, and N‐terminal acetylation were included as variable modifications. A false discovery rate (FDR) threshold of 1% was applied at the peptide level for high‐confidence identifications. For the proteome‐wide comparison, abundance values were normalized to the summed abundance of all detected mitochondrial proteins in that run. For the analysis of complex IV composition, each COX subunit was expressed as a fraction of the summed intensity of the 13 quantified COX subunits in that run. Values were log_2_‐transformed, and each row of the heat map was then scaled to its own mean and standard deviation across the six samples (row‐wise *z*‐score).

### 2.7 Statistical analysis

Statistical analysis of the data was performed using Graphpad Prism v10 (Graphpad Software, San Diego, CA, USA). Comparisons with only two groups, unpaired student's two‐tailed *t*‐test was performed assuming equal variance. The *p*‐values are indicated in the figure legends, where a *p*‐value <0.05 was considered statistically significant. For proteomics data, differential abundance between hypoxia and normoxia was assessed by unpaired two‐tailed Student's *t*‐test assuming equal variance on the log_2_‐transformed, mitochondria‐normalized intensities (*n* = 3 per group). *p*‐values were corrected for multiple testing by the Benjamini‐Hochberg procedure within the 13 quantified complex IV subunits and the pre‐specified hypothesis, and are reported as the false discovery rate (FDR); the comparison across all 329 detected mitochondrial proteins is reported as exploratory.

## 3 RESULTS

Isolation of PASMCs is advantageous to study processes in vitro, such as HPV, in which these cells play an important role. Isolated PASMCs allow direct manipulation of the purified cell population and enable several experimental approaches that are difficult to perform in vivo, such as measurements of Ca^2+^ influx in response to hypoxia. However, obtaining a highly enriched population of PASMCs by manual dissection from distal PAs can be technically challenging and is associated with several limitations. These challenges are partly attributable to the difficulty of reliably identifying and hand‐dissecting distal PAs, especially so for less experienced researchers. To address these limitations, we show that the use of a blue food dye enables rapid and reliable visualization of PAs, greatly facilitating their identification and dissection (Figure 1).

### 3.1 Morphological characterization

Following isolation and initial culture, PASMCs became adherent and began to proliferate approximately 3 days post‐isolation (Figure 3a). Morphological analysis showed that the isolated cells exhibited the elongated, spindle‐shaped morphology of SMCs (Figure 3b,c). Upon reaching confluence, cells displayed the classical “hill‐and‐valley” growth pattern typical for SMCs.

### 3.2 Immunostaining for SMC markers

**FIGURE 4:**
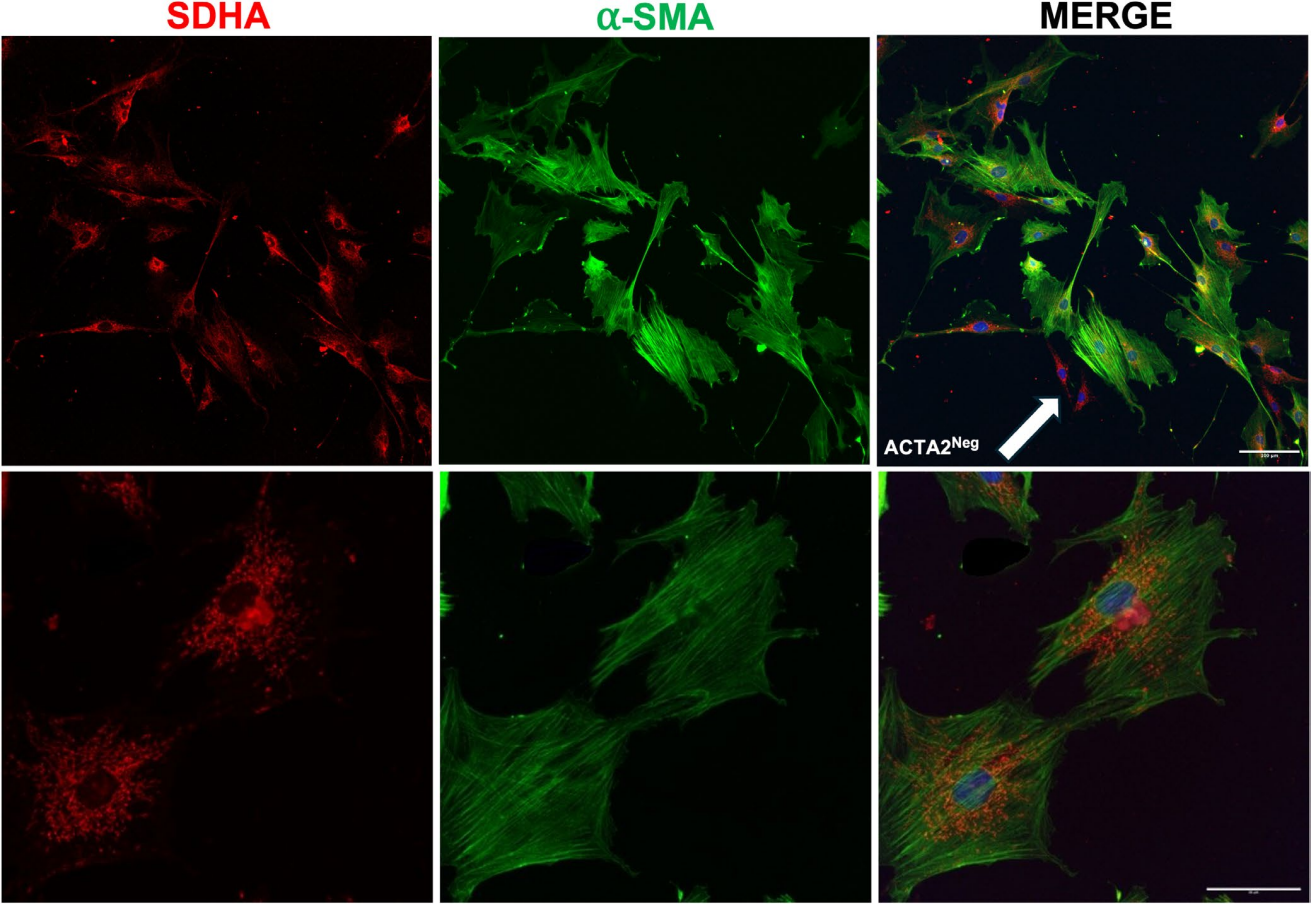
Immunostaining for SMC marker in primary PASMCs. Immunofluorescence staining of rat PASMCs at passage 1 for smooth muscle cell (SMC) marker alpha‐smooth muscle Actin (α‐SMA) in green. Co‐stained mitochondria by SDHA in red. Top panel, 20X; scale bar 100 μm. Bottom panel, 60X; scale bar 50 μm. A total of 3 independent pluck preparations were performed and confirmed. Data presented represents a single isolation.

**FIGURE 5:**
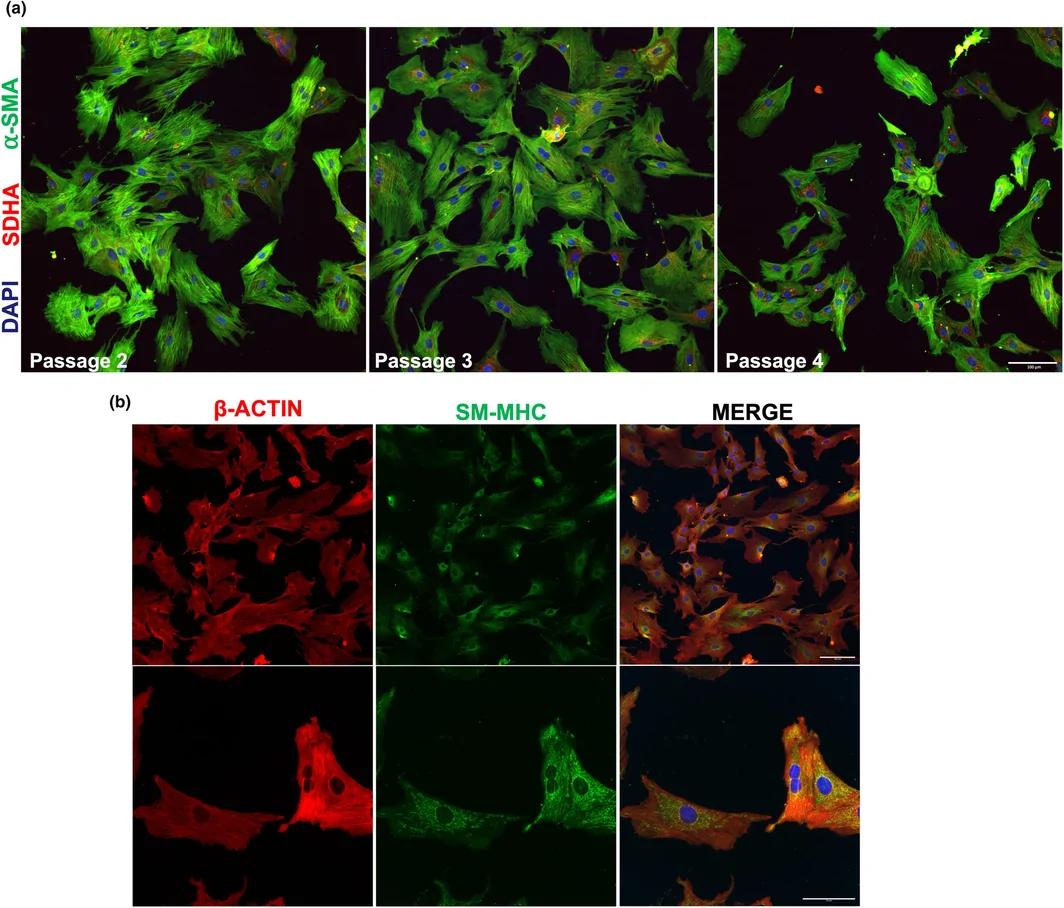
Evaluation of α‐SMA positive cells over time. (a) Immunofluorescence staining of rat PASMCs from passages 2–4 for smooth muscle cell (SMC) marker alpha‐smooth muscle Actin (α‐SMA) in green. Mitochondria were co‐stained with SDHA in red. 20X; scale bar 100 μm. (b) Immunofluorescence staining of rat PASMCs at passage 4 for SMC marker smooth muscle myosin heavy chain (SM‐MHC) in green co‐stained with β‐Actin in red. Top panel, 20X; scale bar 100 μm. Bottom panel, 60X; scale bar 50 μm. A total of 3 independent pluck preparations were performed and confirmed. Data presented represents a single isolation.

Immunofluorescence staining using an antibody against SMC marker alpha‐smooth muscle actin (α‐SMA) was performed to confirm the isolated cells were indeed SMCs, in addition to assessing the overall cell population identities (Figure 4). As a counter‐stain to reveal any potential α‐SMA‐negative cells, an antibody against succinate dehydrogenase complex subunit A (SDHA) was used. Cells were positive for α‐SMA and displayed the cable‐like myofilaments similar to previous reports (Peng et al., 2017), although there were a few that were identified as being α‐SMA‐negative. The isolated cell population was high in α‐SMA ‐positive cells (~92%, 160 positive cells out of 173 cells), indicating the isolation yielded mostly PASMCs. Primary cells in culture are subjected to lose their identities through loss of mechanical, biochemical, and matrix cues of their environment resulting in changes to cell‐type specific markers (Diaz Espinosa et al., 2025). To evaluate if the cells retain their markers over time or if they are outgrown by other cell types, we stained the rat cells in culture for up to 4 passages. The cells remained α‐SMA positive with high purity over time, indicating they retained their identity in culture (Figure 5a). In addition to being a PASMC marker, α‐SMA is also known to be expressed in myofibroblasts. Therefore, to assess whether the cells isolated are contractile SMCs, an additional marker was used, smooth‐muscle myosin heavy chain (SM‐MHC or MYH11). Immunostaining revealed that 90% (144 positive cells out of 160 cells) of the isolated cells were also positive for this marker at passage 4 (Figure 5b). Additionally, we evaluated markers of endothelial cells including Von Willebrand Factor (VWF) and Platelet Endothelial Cell Adhesion Molecule‐1 (PECAM‐1) via RT‐qPCR where the C_T_ values were 32 and 36 respectively (data not shown).

### 3.3 Chronic response of cultured PASMCs to hypoxia induces COX4i2

A known oxygen sensor component required for HPV is complex IV (COX) of the electron transport chain, in particular subunit isoform COX4i2. The expression of this subunit is tissue‐ and cell‐type specific, with high expression in contractile SMCs in the lung, in addition to the carotid body and placenta (Hüttemann et al., 2001). When this gene was knocked out in mice, the HPV response was abolished, indicating its role in oxygen sensing (Sommer et al., 2017). The promoter region of the gene is controlled by an oxygen response element (ORE) and HIF1⍺ (Aras et al., 2013; Fukuda et al., 2007; Hüttemann et al., 2007), and under chronic hypoxic conditions the expression of the gene is increased (Sommer et al., 2017).

**FIGURE 6:**
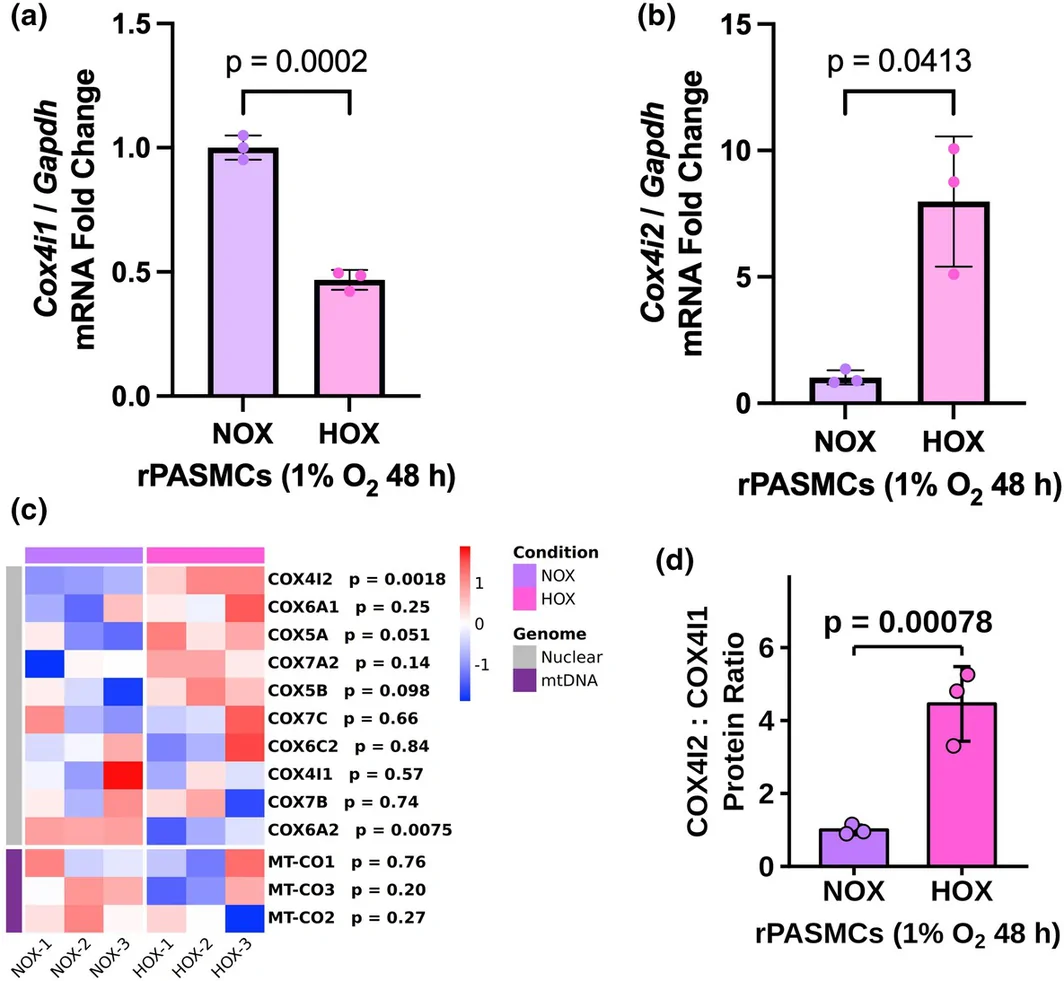
Chronic hypoxia induces Cox4i2 expression in rat PASMCs after cells have been cultured for 4 passages. RT‐qPCR analysis of rat PASMCs under normoxia (NOX) and hypoxia (HOX). (a) *Cox4i1* and (b) *Cox4i2* mRNA fold change following 48 h of hypoxia (1% O_2_). GAPDH was used as a house‐keeping gene. Statistical analysis was performed using an unpaired *t*‐test with *p* values <0.05 were considered to be statistically significant. Data represented as mean ± standard deviation, *n* = 3. (c) Complex IV subunit composition in mitochondria isolated from PASMCs after 48 h at 21% or 1% O_2_. Each of the 13 quantified cytochrome *c* oxidase subunits was expressed as a fraction of the summed intensities of all COX subunits in the same run and log_2_‐transformed; each row was then scaled to its own mean and standard deviation across the six samples (row‐wise *z*‐score). Color therefore indicates how consistently a subunit separates the two conditions and not the magnitude of the change; fold changes are given in Table S1. The ten nuclear‐encoded subunits are shown above the three mitochondrially encoded subunits, each block ordered by fold change from most increased to most decreased under hypoxia. The *p*‐values shown are from an unpaired two‐tailed Student's *t*‐test on the log_2_ values, *n* = 3 preparations per condition. (d) COX4I2:COX4I1 protein ratio, expressed relative to the normoxic mean. Bars are means ± SD with the individual preparations overlaid. Because both isoforms are measured in the same run, the ratio is independent of protein loading and mitochondrial recovery. Unpaired two‐tailed Student's *t*‐test on log_2_ ratios, *n* = 3 preparations per condition.

We here show that *Cox4i2* expression was still present at passage 4, after the cells have been kept in culture for approximately 4 weeks. To confirm the presence of COX4i2 in the isolated cells and their response to hypoxia, we placed the rat PASMCs in hypoxia (1% O_2_) for 48 h and verified an established response to chronic hypoxia, that is, the induction of *Cox4i2* expression, in addition to the presence of the protein. RT‐qPCR showed an 8‐fold induction of *Cox4i2* transcript levels, with a concomitant decrease in transcript levels of *Cox4i1* in PASMCs exposed to 48 h of hypoxia (Figure 6a,b). Mass spectrometry analysis of the isolated rat mitochondria confirmed detectable COX4i2 peptides under normoxia and an about 4‐fold increase after exposure to hypoxia (1% O_2_) for 48 h (4.06‐fold, *p* = 0.0018, FDR = 0.024). Expressed as the COX4I2:COX4I1 ratio, which is independent of protein loading and mitochondrial recovery because both isoforms are measured in the same run, the increase was 4.4‐fold (*p* = 0.00078; Figure 6d). The mass spectrometry data validate the increase in *Cox4i2* transcript levels seen by qPCR. COX4I1 mRNA was downregulated as part of this broader hypoxic reprogramming of transcript stability and translation. But protein levels were unchanged (0.92‐fold, *p* = 0.57), indicating that the decrease in transcript had not yet propagated to the protein level within the 48 h window. COX4i2, in contrast, is oxygen sensitive and its turnover rate is directly correlated to the RNA levels. Among the remaining COX subunits, COX6A2 decreased to 0.11‐fold (*p* = 0.0075, FDR = 0.049), whereas COX5A (1.65‐fold, *p* = 0.051), COX6A1, COX5B, COX6C2, COX7A2, COX7B, and COX7C did not reach statistical significance. The three mitochondrially encoded complex IV subunits showed a trend toward lower abundance (MT‐CO1 0.89‐fold, MT‐CO2 0.61‐fold, MT‐CO3 0.64‐fold) that did not reach statistical significance (Figure 6c).

Taken together, dissecting arterial branches starting from the 3rd and exceeding the 4th generation (with diameters of ~150–300 μm) until the dye was no longer visible yielded cells from the smaller arteries that may be a little larger than precapillary vessels but still expressed *Cox4i2*.

## 4 DISCUSSION

We here describe a fast method for isolation and culture of PASMCs, which exhibit PASMC morphological and cell‐specific markers such as α‐SMA and SM‐MHC. The isolation procedure yielded highly enriched PASMCs expressing markers which remained stable over several passages. The cultured rat PASMCs exhibited a known chronic response to hypoxia, with upregulation of *Cox4i2* transcript expression. This study describes an alternative, low‐cost, and fast method to isolate PASMCs that may help researchers study mechanisms of HPV and pulmonary hypertension in vitro. The use of the food‐grade dye did not affect the cells, and once in culture, the dye is no longer present.

We acknowledge that other methods like magnetic or FACS sorting can yield highly pure cell populations; however, for laboratories that lack these resources, the agar‐dye method described here is intended as a complementary, accessible alternative. By providing a continuous visual guide through the arterials, enabling operators with modest dissection experience to reliably identify and harvest pulmonary arterial segments and to separate different PASMCs stemming from different branching.

COX4I2 expression in primary PASMCs has previously been reported to increase approximately 2‐fold at 4% O_2_ (Aras et al., 2013). In the present study, we observed a greater induction of *Cox4i2*, approximately 8‐fold at the mRNA level and approximately 4‐fold at the protein level, under 1% O_2_. This robust induction is consistent with the distal arterial identity of the cells isolated by our method, as COX4I2 expression is a key molecular feature of PASMCs required for HPV (Hüttemann et al., 2001; Sommer et al., 2017). Unlike many nuclear‐encoded mitochondrial subunits, COX4I2 is regulated in a HIF‐1α‐independent manner through a conserved oxygen‐responsive element in its proximal promoter (Aras et al., 2013). In contrast, *Cox4i1* mRNA was reduced under hypoxia, yet COX4I1 protein levels remained stable as judged by mass spectrometry. This mRNA‐protein discordance suggests that the pre‐existing pool of COX4I1 already incorporated into assembled complex IV turns over slowly, so that the reduction in transcript had not yet produced a change detectable at the protein level within our experimental window. Notably, mass spectrometry data also revealed a trend toward reduced detection of the three mitochondrially encoded core subunits of complex IV, MT‐CO1, MT‐CO2 and MT‐CO3, after hypoxia, which did not reach statistical significance. This observation is consistent with emerging evidence demonstrating coordinated downregulation of mitochondrially encoded OXPHOS transcripts under hypoxia through other mechanisms distinct from the nuclear‐encoded OXPHOS response (Shtolz et al., 2026). Taken together, these findings suggest a shift in complex IV composition toward a higher proportion of COX4I2‐containing isoforms under chronic hypoxia. COX4I2 shows a direct correlation between mRNA and protein levels, making it an easy to use marker for validating PASMC's successful induction of hypoxia.

Future studies employing this isolation technique could systematically compare COX4i2 expression between freshly isolated and cultured PASMCs, and between proximal and distal arterial segments, to define the COX4i2 expression gradient along the pulmonary arterial tree and to quantify phenotypic drift associated with in vitro culture.

## AUTHOR CONTRIBUTIONS

**Lucynda Pham:** Data curation; formal analysis; investigation; methodology. **Lauren Pavelich:** Formal analysis; investigation. **Natascha Sommer:** Formal analysis; investigation. **Lawrence I. Grossman:** Formal analysis; investigation. **Maik Hüttemann:** Conceptualization; formal analysis; investigation; methodology; supervision. **Tasnim Arroum:** Conceptualization; data curation; formal analysis; investigation; methodology; supervision.

## FUNDING INFORMATION

This work was supported by NSF grant MCB‐2329629 and the German Research Society (DFG), project number 521904638. The authors gratefully acknowledge support from the Corrine F. Resta Endowed Doctoral Support Award (Wayne State University) and the Thomas C. Rumble University Graduate Fellowship (Wayne State University).

## CONFLICT OF INTEREST STATEMENT

The authors declare no conflicts of interest, financial, or otherwise.

## ETHICS STATEMENT

All animal tissue used in this study was obtained under Wayne State University's Institutional Animal Care and Use Committee (IACUC) protocol 22‐04‐4589. Heart‐lung plucks from male Sprague Dawley rats (age 6 months; *n* = 3) were collected as discarded tissue immediately after the animals were euthanized with an overdose of sodium pentobarbital (200 mg/kg, i.p.). No animals were euthanized specifically for this study. All procedures were conducted in accordance with the NIH Guide for the Care and Use of Laboratory Animals. This study did not involve human participants, human tissue, or human data.

## Supporting information


**Figure S1.** Exploratory comparison of the mitochondrial proteome between normoxia and hypoxia. Volcano plot of all 329 mitochondrial proteins quantified in all six preparations. Abundances were expressed as a fraction of the summed mitochondrial intensity in the same run and log_2_‐transformed; fold change is hypoxia relative to normoxia. Points are colored where an unpaired two‐tailed Student's *t*‐test on the log_2_ values reached *p* < 0.05 (*n* = 3 preparations per condition); the dashed line marks *p* = 0.05, and 43 proteins fall above it against 16 expected by chance. No protein survived Benjamini‐Hochberg correction across the 329 proteins (lowest FDR 0.097), so this comparison is exploratory and individual proteins should not be interpreted as significantly changed.

**Table S1.** Mitochondrial proteomics of rat pulmonary arterial smooth muscle cells: normoxia versus chronic hypoxia. (A) COX4I2 fold change across normalizations, (B) Complex IV subunit fold changes, (C) Mitochondrial proteins—normalized abundance, (D) Mitochondrial proteome statistics, and (E) Proteins with *p* < 0.05 (exploratory).

## Data Availability

The data and [Link], [Link] are available within the article. The mass spectrometry proteomics data have been deposited to the ProteomeXchange Consortium via the PRIDE partner repository with the dataset identifier PXD083383.
