# Supplementary material for: Arterial tracing‐based technique for rapid purification of pulmonary arterial smooth muscle cells from rat lungs
Source: Physiol Rep. 2026 Sep 7;14(17):e71098. doi: 10.14814/phy2.71098 (PMC13550767; doi:10.14814/phy2.71098)
Supplement: Supplementary file 1 [file PHY2-14-e71098-s001.pdf]

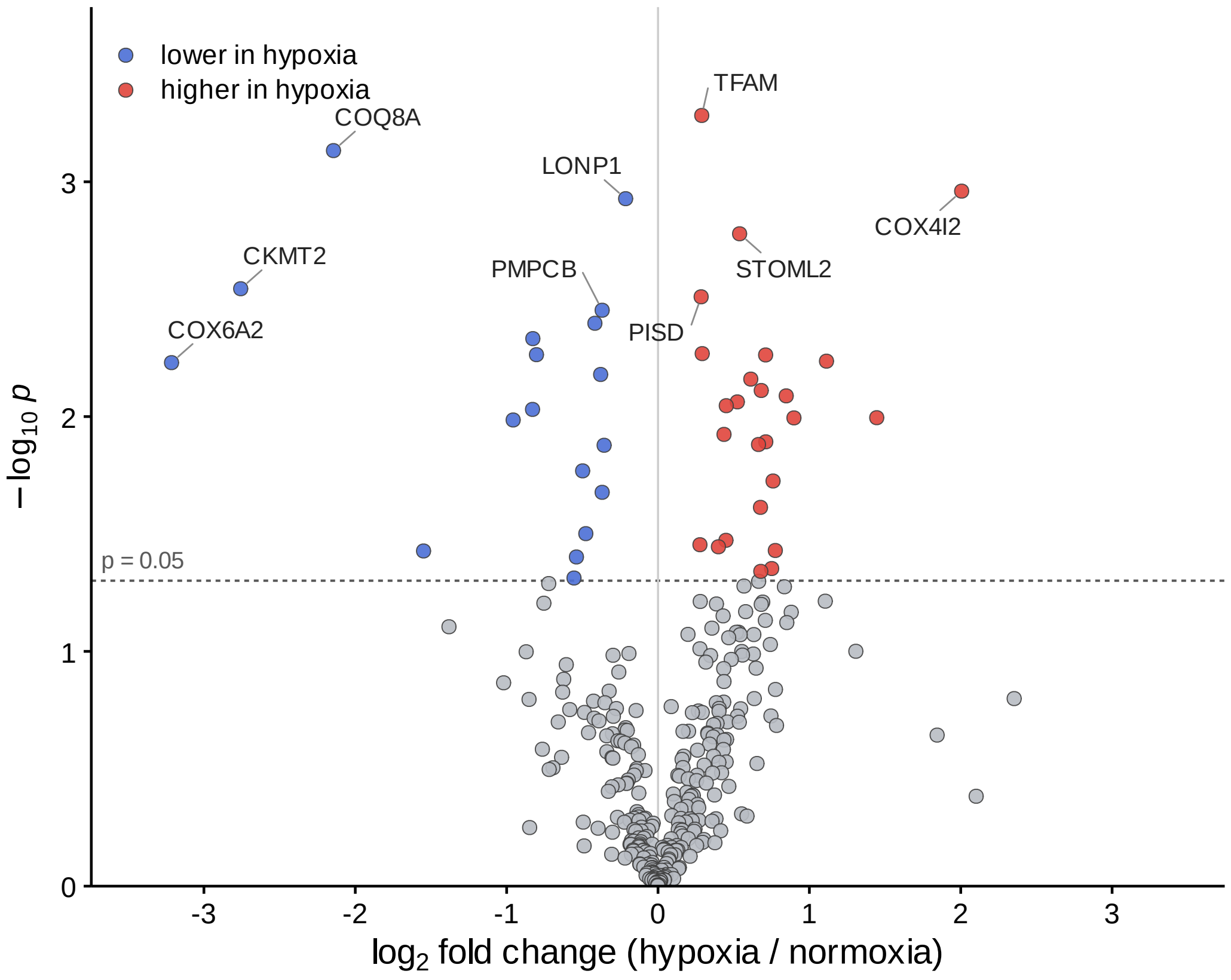

lower in hypoxia
higher in hypoxia
COQ8A
TFAM
LONP1
COX4I2
CKMT2
PMPCB
STOML2
COX6A2
PISD
p = 0.05
$-\log_{10} p$
$\log_2$ fold change (hypoxia / normoxia)
0
1
2
3
-3
-2
-1
0
1
2
3
