# Supplementary material for: Arterial tracing‐based technique for rapid purification of pulmonary arterial smooth muscle cells from rat lungs
Source: Physiol Rep. 2026 Sep 7;14(17):e71098. doi: 10.14814/phy2.71098 (PMC13550767; doi:10.14814/phy2.71098)
Supplement: Supplementary file 2 [file PHY2-14-e71098-s002.pdf]

Supplemental Table S1

Mitochondrial proteomics of rat pulmonary arterial smooth muscle cells: normoxia versus chronic hypoxia

A. COX4I2 fold change across normalizations

COX4I2 fold change (hypoxia vs normoxia) under seven denominators, showing the increase does not depend on the choice of normalization.

Normalization	FoldChange	p_value
COX4I2 : COX4I1 (direct ratio)	4.4	0.00078
COX4I2 / total mitochondrial signal	4.02	0.0011
COX4I2 / total protein	3.83	0.00152
COX4I2 / sum of all 13 COX subunits	4.06	0.00184
COX4I2 / sum of the other 12 COX subunits	4.07	0.00186
COX4I2 / MT-CO1+2+3	5.91	0.00619
COX4I2 raw intensity (no denominator)	3.78	0.00166

B. Complex IV subunit fold changes

Fold change of each complex IV subunit as a share of the summed COX signal (basis of Figure 6C). FDR is Benjamini-Hochberg within the 13 subunits.

Subunit	Genome	FoldChange	p_value	FDR_within_COX
COX4I2	Nuclear	4.056	0.001844	0.02397
COX6A1	Nuclear	3.624	0.2486	0.4418
COX5A	Nuclear	1.65	0.05059	0.2192
COX7A2	Nuclear	1.465	0.1429	0.3715
COX5B	Nuclear	1.302	0.09828	0.3194
COX7C	Nuclear	1.122	0.6641	0.8245
COX6C2	Nuclear	0.929	0.8426	0.8426
COX4I1	Nuclear	0.923	0.5686	0.8213
COX7B	Nuclear	0.901	0.7398	0.8245
MT-CO1	mtDNA	0.893	0.7611	0.8245
MT-CO3	mtDNA	0.639	0.205	0.4418
MT-CO2	mtDNA	0.613	0.2719	0.4418
COX6A2	Nuclear	0.109	0.007532	0.04896

C. Mitochondrial proteins - normalized abundance

Abundance of each mitochondrial protein quantified as a fraction of the summed mitochondrial signal in each sample. Values are on a linear scale, and each column sums to 1.

Accession	Gene	NOX-1	NOX-2	NOX-3	HOX-1	HOX-2	HOX-3
A0A0G2K047	Acss3	5.006e-05	5.635e-05	6.777e-05	3.066e-05	3.227e-05	3.64e-05
A0A0G2K309	Slc25a15	0.00017141	0.00014488	0.00015664	0.00012279	0.00011737	0.00012262
A0A0G2K5L2	Slc25a22	0.00097928	0.0010654	0.00075136	0.00124779	0.00109492	0.00078779
A0A8I6A2H6	Micu3	6.074e-05	9.78e-05	3.742e-05	5.479e-05	5.073e-05	5.712e-05
A1L1L6	Rhot1	0.00047224	0.00083768	0.00085993	0.00108813	0.0011772	0.00079164
A2VCW9	Aass	0.0001092	8.893e-05	0.00012475	6.028e-05	8.557e-05	9.688e-05
A2VD13	Ptcd1	2.268e-05	1.626e-05	5.943e-05	3.358e-05	2.193e-05	2.654e-05
A4F267	Tomm40l	0.000187	0.00035452	0.00057541	0.00056226	0.00054301	0.00058904
B0BN56	Mrps31	0.00059064	0.00066558	0.00049231	0.0006201	0.00062825	0.00066628
B0BN86	Tmem11	0.00144416	0.00085616	0.00113319	0.00149217	0.00155675	0.00148788
B0K036	Sdhaf1	0.00024078	0.00039431	0.0001857	9.34e-05	0.00032146	0.00025778
B1WC37	Trmu	0.00024775	0.00022692	0.00024328	0.00020471	0.00022535	0.00028347
B1WC61	Acad9	0.00269041	0.00315273	0.00280897	0.00350146	0.00276951	0.00322755
B2GV06	Oxct1	0.00756717	0.00763948	0.00925951	0.00624866	0.00894971	0.0123229
B2GV71	Ndufaf5	0.00016097	0.00018241	7.971e-05	0.00015305	0.00015453	0.00021508
B2RYW9	Fahd2a	0.00112543	0.00116646	0.00086034	0.00136154	0.00104577	0.00082386
B2RZD2	Tmem126b	5.732e-05	9.6e-05	1.793e-05	2.202e-05	3.935e-05	6.03e-05
B5DF07	Prorp	3.972e-05	5.426e-05	2.814e-05	5.138e-05	5.211e-05	5.1e-05
D3Z9R8	Atp5mpl	0.00263953	0.00248605	0.00073721	0.0022117	0.00200604	0.00200947
D3ZAF6	Atp5mf	0.00259143	0.00182558	0.00195132	0.00472162	0.00378467	0.00523012
D3ZAW2	Pisd	0.00019818	0.00017937	0.00018918	0.00023037	0.00022538	0.0002344
D3ZKT0	Tamm41	0.00024591	0.00031675	0.00033592	0.00031362	0.00026404	0.00038131
D3ZN43	Ndufaf6	7.453e-05	7.781e-05	5.517e-05	6.971e-05	4.658e-05	0.00011102
D3ZS74	Oma1	8.501e-05	0.00011861	0.00015242	0.00013577	0.00010255	0.00012339
D3ZUA0	Mthfd2l	0.0001666	0.00012264	2.869e-05	0.00012093	0.00010545	0.00015623
D3ZX08	Aars2	0.00027914	0.00029396	0.00021563	0.00021926	0.0002372	0.00021825
D3ZYW7	Fxn	0.00011095	0.00011409	0.00010697	0.00017581	0.00015361	0.00024348
D4A6D7	Ttc19	4.991e-05	4.78e-05	6.258e-05	0.0001024	6.209e-05	0.00013769
F1LU71	Auh	0.00117146	0.00127667	0.00083034	0.00109282	0.00101514	0.00102447
F1LX07	Slc25a12	0.00223896	0.00191222	0.00174367	0.0019409	0.00187791	0.00132718
F1LZW6	Slc25a13	0.00204995	0.00172996	0.00173782	0.00194921	0.00160428	0.00165075
G3V9A7	Mtfr1	6.729e-05	2.591e-05	0.00014721	0.0001183	7.646e-05	6.198e-05
O08776	Ndufaf3	0.00031	0.00024535	0.00052013	0.0003217	0.00025633	0.00012761
O35093	Timm23	0.00021088	0.00031281	0.00012439	0.00014258	0.00017725	0.00025267
O35094	Timm44	0.00109761	0.00121786	0.00121368	0.00122797	0.00104787	0.00116234
O35796	C1qbp	0.00206637	0.00250924	0.00404998	0.00206316	0.00221591	0.0034508
O35854	Bcat2	0.00247099	0.00213452	0.00335779	0.00204798	0.00238262	0.00294866
O35952	Hagh	0.00042133	0.000541	0.00039559	0.00046667	0.00044475	0.00069914
O55171	Acot2	0.00166911	0.00147095	0.00205004	0.00167552	0.00115135	0.00139553

Accession	Gene	NOX-1	NOX-2	NOX-3	HOX-1	HOX-2	HOX-3
O88426	Nme6	0.00013413	9.764e-05	0.00012532	0.00012089	0.00014534	0.00023952
O88483	Pdp1	0.00034393	0.00048464	0.00042788	0.00078173	0.00065413	0.00081148
O88484	Pdp2	5.983e-05	7.113e-05	0.00010794	9.543e-05	8.856e-05	0.00010258
O88884	Akap1	0.00024914	0.0002659	0.00018811	0.00029604	0.00029064	0.00027955
O88994	Mtarc2	0.00186233	0.00181654	0.00140648	0.00239256	0.00223026	0.00198941
O89035	Slc25a10	0.00083861	0.00088043	0.00066331	0.0008527	0.00067312	0.00049398
P00159	Mt-cyb	0.0002996	0.00034822	0.00059598	0.00074708	0.00072593	0.00065092
P00406	Mt-co2	0.0110771	0.0197398	0.00919712	0.0127262	0.00955438	0.00371064
P00507	Got2	0.0134497	0.0118648	0.00796443	0.0128725	0.0114431	0.0135689
P03889	Mt-nd1	0.00133885	0.00056926	0.00099418	0.00079892	0.00067919	0.00079992
P04182	Oat	0.0061011	0.00566096	0.00837142	0.00855674	0.00621664	0.00717716
P04636	Mdh2	0.0248139	0.0253101	0.0247514	0.0263501	0.0235302	0.0231544
P05503	Mt-co1	0.00162174	0.00103744	0.00088195	0.00091201	0.00066307	0.00170095
P05504	Mt-atp6	0.00012584	0.00025281	0.00016057	0.00023512	0.00018822	0.00019842
P05505	Mt-co3	0.00243793	0.00428067	0.00305912	0.00150782	0.00163007	0.00329818
P05508	Mt-nd4	0.00230088	0.00203014	0.00115528	0.00174357	0.00157136	0.00141513
P07633	Pccb	0.00079619	0.00095058	0.00092945	0.00080002	0.00073432	0.0008034
P07756	Cps1	0.00030681	0.00041418	0.0010064	0.00023858	0.00031699	0.00020256
P07895	Sod2	0.00570846	0.00358116	0.00571619	0.00723328	0.00553315	0.00593448
P08461	Dlat	0.00990766	0.00851396	0.00742322	0.00847431	0.00710829	0.00658633
P08503	Acadm	0.00351326	0.00356695	0.00298254	0.00481456	0.0038564	0.00461832
P09605	Ckmt2	0.00614863	0.00446292	0.00270556	0.00045815	0.00063787	0.00082317
P0C2B9	Mrpl42	0.00011675	0.00010777	0.00011159	0.00014889	0.0001074	0.00019738
P0C2C0	Mrpl22	0.00041715	0.00042827	0.00058673	0.00042418	0.0002373	0.00037893
P0C2C1	Mrpl30	0.00024043	0.00023837	0.00041426	0.00040214	0.00030993	0.00040867
P0C2C4	Mrpl10	0.00015876	0.000135	0.00026787	0.00014804	0.00011439	0.00017155
P0C2X9	Aldh4a1	0.0008516	0.00091621	0.00174872	0.00061595	0.00071782	0.00083346
P0C546	Slc25a42	0.00022745	0.00023979	0.00056981	0.00033529	0.00026219	0.00066392
P0DKR2	Tcaim	3.692e-05	4.302e-05	3.2e-05	5.264e-05	3.758e-05	3.812e-05
P0DN35	Ndufb1	0.00017954	0.00019246	0.00022223	0.00023366	0.0003027	6.904e-05
P10719	Atp5f1b	0.0405795	0.0443326	0.0554324	0.0314257	0.0413354	0.0391917
P10817	Cox6a2	0.00038512	0.00040107	0.00035035	1.918e-05	4.408e-05	8.013e-05
P10818	Cox6a1	9.028e-05	5.216e-05	0.00047033	0.0003298	0.0002039	0.00152386
P10860	Glud1	0.0132334	0.0166866	0.0160522	0.0220797	0.0226825	0.0253385
P10888	Cox4i1	0.0162688	0.0170249	0.020037	0.0158404	0.0172586	0.0155002
P11240	Cox5a	0.00766784	0.00592663	0.00424137	0.0115551	0.0078874	0.00923411
P11608	Mt-atp8	0.00040876	0.00060142	0.00043244	0.00054729	0.00047004	0.00036123
P11661	Mt-nd5	0.00320854	0.00330528	0.00341514	0.00326286	0.00271776	0.00376569
P11662	Mt-nd2	1.751e-05	6.7e-06	0.00023815	1.247e-05	0.00061148	0.00028977
P11884	Aldh2	0.0138506	0.0117716	0.0151324	0.00928569	0.0133551	0.0152687
P11951	Cox6c2	0.0024167	0.00316162	0.00340212	0.00194211	0.00210143	0.00496941
P11960	Bckdha	0.00222184	0.00234163	0.00163402	0.00208019	0.00194891	0.00186147

Accession	Gene	NOX-1	NOX-2	NOX-3	HOX-1	HOX-2	HOX-3
P12007	Ivd	0.0035379	0.00400701	0.00265402	0.00239892	0.0022087	0.00231268
P12075	Cox5b	0.00963347	0.0102163	0.00607628	0.0107048	0.0116279	0.0102959
P13086	Suclg1	0.00356152	0.00315674	0.00510076	0.00719014	0.00492884	0.00682862
P13264	Gls	0.00980677	0.0098732	0.00664197	0.0133218	0.0136579	0.0144599
P13264-2	Gls	0.00203196	0.00183748	0.00194914	0.00260115	0.00329675	0.00350337
P13437	Acaa2	0.00169821	0.00227344	0.00343008	0.0012644	0.00160731	0.00178541
P13803	Etfa	0.00547586	0.0049099	0.00497586	0.00676864	0.00694737	0.00495591
P14408-2	Fh	0.0042339	0.00431895	0.00518447	0.00478638	0.00431366	0.00472402
P14604	Echs1	0.00310594	0.00290893	0.00372105	0.0032766	0.00294128	0.00267851
P14882	Pcca	0.00106968	0.00101058	0.00116293	0.00128376	0.00103269	0.0011691
P15650	Acadl	0.00515354	0.00468041	0.00430628	0.00597995	0.0068588	0.00464521
P15651	Acads	0.00137979	0.00133251	0.0016167	0.00115166	0.0015746	0.00163369
P15999	Atp5f1a	0.0568995	0.0548701	0.0478331	0.0522732	0.0497506	0.0567827
P16036	Slc25a3	0.0354785	0.0378397	0.0357711	0.0423145	0.0388756	0.0300894
P16261	Slc25a16	0.00012597	0.00013976	0.00015843	0.00012706	0.00015948	0.00021055
P17178	Cyp27a1	0.00018313	0.00020238	0.00018262	0.00012357	0.00010977	8.941e-05
P17764	Acat1	0.00663595	0.00689892	0.00629223	0.00714885	0.00732076	0.00599858
P18665	Mrpl3	0.00048383	0.00072231	0.0011169	0.00056249	0.00062591	0.00091258
P18886	Cpt2	0.00182185	0.00157985	0.00157012	0.00149286	0.00150199	0.00176438
P19234	Ndufv2	0.00413415	0.00405598	0.00323646	0.00435224	0.00346901	0.00360292
P19511	Atp5pb	0.00708382	0.00780982	0.0105477	0.0124066	0.00991177	0.0146824
P20069	Pmpca	0.00065793	0.00066594	0.00041368	0.00063697	0.00060724	0.00054363
P20788	Uqcrfs1	0.00794808	0.00919738	0.0106918	0.00682349	0.0077801	0.00794645
P21571	Atp5pf	0.00106197	0.00144698	0.00126508	0.00151276	0.0021098	0.00242684
P21913	Sdhb	0.00409685	0.0031434	0.00335932	0.00335018	0.00463575	0.00479432
P22791	Hmgcs2	6.397e-05	5.994e-05	0.00020758	4.696e-05	6.77e-05	5.908e-05
P23965	Eci1	0.00439453	0.00389741	0.00469986	0.0066378	0.00560083	0.00643347
P24483	Fdx1	0.00045783	0.00065368	0.00035324	0.00059638	0.00051466	0.00050389
P26284	Pdha1	0.00606156	0.00615973	0.00420538	0.0064477	0.00554478	0.00602353
P26772	Hspe1	0.0393035	0.034258	0.0452423	0.0291626	0.0426961	0.0263098
P28042	Ssbp1	0.00224978	0.0027122	0.00168931	0.00304421	0.0035527	0.00166976
P29117	Ppif	0.00065513	0.00069289	0.00048015	0.00057855	0.00058362	0.00049686
P29147	Bdh1	0.0020127	0.00203857	0.00181255	0.0045908	0.00317178	0.00255832
P29266	Hibadh	0.00262046	0.0027182	0.00235969	0.00268527	0.00211424	0.00221361
P29410	Ak2	0.00142319	0.00150904	0.00206907	0.00214967	0.00209577	0.00379725
P29411	Ak3	0.00834419	0.00911968	0.00584084	0.011102	0.00854683	0.00796819
P29418	Atp5f1e	0.00165161	0.00052897	9.659e-05	0.00251716	0.00096288	0.00463791
P29419	Atp5me	0.00228925	0.00253046	0.00372424	0.00289015	0.0019947	0.00254558
P31399	Atp5pd	0.00552238	0.00666209	0.0104856	0.0105444	0.00797966	0.0113518
P32089	Slc25a1	0.00424961	0.00352355	0.00577743	0.00694341	0.00677365	0.00556208
P32551	Uqcrc2	0.00931829	0.00795943	0.0110055	0.00827941	0.00792045	0.00896292
P35171	Cox7a2	0.00049826	0.00107981	0.00082713	0.00125289	0.0011582	0.00093653

Accession	Gene	NOX-1	NOX-2	NOX-3	HOX-1	HOX-2	HOX-3
P35434	Atp5f1d	0.00553693	0.0068874	0.00642281	0.00667989	0.0111925	0.0089662
P35435	Atp5f1c	0.00621113	0.00707456	0.00607253	0.00782685	0.00592764	0.0065478
P35571	Gpd2	0.00045308	0.0004358	0.00045372	0.0005479	0.00060936	0.00068491
P35738	Bckdhb	0.00099137	0.00135901	0.00354381	0.00088745	0.00085212	0.0012891
P38718	Mpc2	0.00307419	0.00295078	0.00199326	0.00588157	0.00309206	0.00168861
P41565	Idh3g	0.00329941	0.0035851	0.00319601	0.00285418	0.00264889	0.00232289
P42676	Nln	0.0005708	0.00068792	0.00064403	0.00060024	0.00073473	0.00060303
P45953	Acadvl	0.00274244	0.00248557	0.0029369	0.0017373	0.0018452	0.00221607
P48721	Hspa9	0.0193091	0.0200249	0.0212196	0.0140666	0.0151638	0.0161592
P49432	Pdhb	0.00558756	0.00479743	0.00298418	0.00411117	0.00471801	0.00286502
P51650	Aldh5a1	6.016e-05	6.249e-05	0.00014519	3.114e-05	5.194e-05	1.911e-05
P52504	Ndufs6	0.00103638	0.00099479	0.0026922	0.00225609	0.00186464	0.00088445
P52873	Pc	0.00251666	0.00241368	0.00228565	0.00234856	0.00227654	0.00242758
P56522	Fdxr	0.00055662	0.00056971	0.0004256	0.00052669	0.00049535	0.00041267
P56571		0.00347685	0.00332087	0.00344545	0.00414849	0.00314714	0.00281701
P56574	Idh2	0.00996862	0.00994628	0.0110195	0.0107698	0.00904637	0.0109209
P62074	Timm10	4.961e-05	8.993e-05	4.002e-05	0.00015681	0.00012927	8.763e-05
P62076	Timm13	0.00011911	0.0001186	0.00018669	0.0001178	0.00016971	0.00016705
P63031	Mpc1	0.00075154	0.00123034	0.00074451	0.00118955	0.00121068	0.00152375
P63039	Hspd1	0.0350468	0.0318095	0.0390721	0.0188008	0.0282778	0.0258353
P70584	Acadsb	0.00101843	0.00101697	0.00117733	0.00091821	0.00114335	0.00133467
P80431	Cox7b	0.00157167	0.00139699	0.00191628	0.00180389	0.00193462	0.0008565
P80432	Cox7c	0.00230661	0.00169253	0.00120924	0.00162042	0.00157663	0.00254039
P83565	Mrpl40	0.00040916	0.00031851	0.00028157	0.00037225	0.00027793	0.00035552
P84817	Fis1	0.00075238	0.00079411	0.00076031	0.00098863	0.00077592	0.00059085
P84850	D2hgdh	0.00027886	0.0002363	0.00020095	0.00025859	0.00019281	0.00021093
P85834	Tufm	0.00730308	0.00769349	0.00918248	0.00655857	0.0073923	0.00761811
P97519	Hmgcl	0.00096895	0.00108297	0.00139759	0.00095193	0.00163871	0.00122142
P97521	Slc25a20	0.00563742	0.00725331	0.00633061	0.00509736	0.00569742	0.00618248
P97564	Gpam	0.00010252	8.242e-05	8.671e-05	0.00011641	0.00010783	0.00018422
P97576	Grpel1	0.00339731	0.00341343	0.00299941	0.00309421	0.00376976	0.0042063
P97615	Txn2	0.00010574	0.00020361	0.00033657	0.00020621	3.753e-05	0.00033946
P97700	Slc25a11	0.00298138	0.00394379	0.00390074	0.00524557	0.00469792	0.00473925
Q01205	Dlst	0.00756754	0.00895963	0.00371846	0.00953386	0.0067358	0.00428385
Q01992	Mipep	0.00026093	0.00022058	0.00011187	0.00020068	0.00020455	0.00023785
Q02253	Aldh6a1	0.00300923	0.00419443	0.00493539	0.00303403	0.00340063	0.00463919
Q03344	Atp5if1	0.00295935	0.00355051	0.00284531	0.0036064	0.00163542	0.00348408
Q03346	Pmpcb	0.00154419	0.00141638	0.00146177	0.00107595	0.00114423	0.00120589
Q05962	Slc25a4	0.0992939	0.0975692	0.0756395	0.0857861	0.08808	0.0795901
Q06645	Atp5mc1	0.00094275	0.00083064	0.0010789	0.00129861	0.00079974	0.00157794
Q06647	Atp5po	0.00960285	0.00996807	0.0107172	0.0102355	0.0109252	0.00974871
Q07116	Suox	0.0001413	0.00016911	0.00017653	9.007e-05	7.666e-05	0.00010894

Accession	Gene	NOX-1	NOX-2	NOX-3	HOX-1	HOX-2	HOX-3
Q07803	Gfm1	0.00058228	0.00051005	0.000546	0.00067272	0.00058749	0.00073331
Q09073	Slc25a5	0.0215921	0.0210232	0.0226028	0.0290548	0.0201121	0.0166452
Q1HCL7	Nadk2	0.00013213	0.00014104	0.0001031	0.00018276	0.00012827	0.00012149
Q2TA68-3	Opa1	0.00153512	0.00190165	0.00158581	0.00169216	0.00144529	0.00197929
Q3B7D0	Cpox	0.00046127	0.00049263	0.00024567	0.00050755	0.0004707	0.00057325
Q3B8P0	Parl	0.0002434	0.00015233	0.00022722	0.00023151	0.00019059	0.00022199
Q3KRD0	Dars2	0.00014475	0.00021828	0.00016857	0.00017922	0.00016444	0.00013003
Q3KRD5	Tomm34	0.00016726	0.00023285	0.00029161	0.00033386	0.00021257	0.00021005
Q3MIE0	Echdc3	0.0002573	0.00022589	0.00036984	0.00031345	0.00016982	0.00037468
Q498T4	Mrpl2	0.00043771	0.00049459	0.00037877	0.00037318	0.00041674	0.00045152
Q498U3	Slc25a40	0.00016538	0.00015316	0.00013465	0.00015401	0.00023201	0.00013052
Q499N4	Coq2	0.00016339	9.476e-05	6.87e-05	9.007e-05	9.37e-05	0.00014034
Q499N5	Acsf2	0.00142543	0.00182357	0.00118099	0.00215479	0.00161158	0.00123764
Q499U1	Slc25a38	1.011e-05	3.514e-05	4.141e-05	2.502e-05	1.882e-05	1.119e-05
Q4FZT0	Stoml2	0.00319784	0.00302177	0.00338412	0.00444671	0.00500404	0.00450726
Q4FZU1	Coq4	0.00011882	8.135e-05	0.00011287	6.932e-05	7.063e-05	0.0001185
Q4FZX5	Msrb2	0.00015078	0.0002445	0.00022324	0.00027856	0.00022409	0.00022585
Q4G064	Coq5	9.333e-05	0.00011191	9.955e-05	8.899e-05	0.00011382	9.237e-05
Q4G078	Mterf4	2.475e-05	1.582e-05	4.673e-05	1.804e-05	2.654e-05	3.51e-05
Q4KM51	Tefm	0.00010031	0.000113	6.812e-05	6.51e-05	8.306e-05	6.347e-05
Q4KM98	Mff	0.00014827	0.00011689	0.00018748	0.00012582	0.00021916	0.00014867
Q4QQT0	Rpusd4	4.176e-05	7.084e-05	4.807e-05	6.623e-05	7.803e-05	6.368e-05
Q4QR80	Mrps25	0.00051197	0.00074067	0.00048029	0.00044459	0.00059663	0.00054127
Q4V7A8	Abhd18	1.375e-05	8.07e-06	3.4e-05	1.746e-05	1.329e-05	2.157e-05
Q4V7E5	Mtrf1l	9.971e-05	0.00012578	0.00013761	0.00011299	8.759e-05	0.00016626
Q4V7F3	Osgepl1	9.023e-05	7.216e-05	6.132e-05	6.06e-05	8.015e-05	8.493e-05
Q4V897	Ccdc90b	0.00039403	0.0003789	0.00045803	0.0004845	0.00046315	0.00045976
Q505J9	Atad1	0.00095484	0.00110634	0.00140883	0.00114987	0.00098956	0.00099924
Q52KK3	Slc25a51	0.00041959	0.0004492	0.00025559	0.00043843	0.00038065	0.00040189
Q561S0	Ndufa10	0.00309268	0.00361675	0.00325847	0.00282513	0.00287224	0.00420011
Q562B5	Pgam5	0.00216353	0.00265362	0.00154822	0.00263703	0.00264091	0.00252579
Q5BJP6	Gfm2	0.00012965	8.703e-05	0.00012497	0.00014074	9.31e-05	0.00011312
Q5BJQ0	Coq8a	0.00025083	0.0002137	0.00033846	7.042e-05	5.379e-05	5.55e-05
Q5BJX1	Mrpl41	0.00083649	0.00041355	0.00032572	0.00057359	0.00041906	0.00051599
Q5BK62	Mpv17	0.00035162	0.00031917	0.00018724	0.00040647	0.00044875	0.00013839
Q5BK63	Ndufa9	0.00225888	0.00195003	0.00203294	0.00196371	0.00195938	0.00172205
Q5EB62	Slc25a46	7.97e-05	8.092e-05	0.00011241	7.735e-05	8.249e-05	0.00010513
Q5EBA0	Eral1	3.825e-05	4.337e-05	4.224e-05	2.583e-05	2.905e-05	4.625e-05
Q5EBA1	Supv3l1	0.0004911	0.00046316	0.00022582	0.00037353	0.0003575	0.00026169
Q5FWT5	Qrsl1	3.846e-05	3.628e-05	5.189e-05	2.965e-05	3.544e-05	1.954e-05
Q5HZE0	Slc25a29	8.179e-05	6.76e-05	7.744e-05	8.245e-05	7.471e-05	5.715e-05
Q5I0C3	Mccc1	0.00084038	0.00102969	0.00079611	0.00064898	0.00069565	0.00086159

Accession	Gene	NOX-1	NOX-2	NOX-3	HOX-1	HOX-2	HOX-3
Q5I0C5	Mtfmt	4.317e-05	6.92e-05	1.397e-05	4.847e-05	4.964e-05	4.104e-05
Q5I0I9	Coq10b	4.55e-05	7.589e-05	5.045e-05	8.119e-05	7.036e-05	7.557e-05
Q5I0K3	Clybl	0.00014984	0.00013509	0.00015768	0.00010324	9.16e-05	0.00012523
Q5I0K5	Abhd10	0.00043075	0.00015472	7.738e-05	0.00030231	0.00022263	0.00024172
Q5I0K8	Mrps7	0.00037775	0.00029741	0.00031747	0.00040061	0.00036287	0.00043692
Q5I0L3	Yars2	0.00026684	0.00022692	0.00017165	0.0002535	0.00022028	0.00015294
Q5I0P2	Gcsh	0.00057575	0.00037885	0.00045726	0.0005352	0.00080003	0.00086876
Q5M7V7	Fastkd2	0.00016744	0.0001505	0.00013632	0.00015549	0.0001566	0.00015196
Q5M7W7	Pars2	0.00014182	0.00015678	0.00015379	0.00017443	9.869e-05	0.00014633
Q5M818	Mrpl16	0.00022746	0.00026702	0.00028905	0.00026616	0.00034817	0.0004669
Q5M9I5	Uqcrh	0.00034305	0.00028241	0.00018043	0.00029147	0.00038119	0.00025153
Q5PPN7	Ccdc51	0.00043154	0.00048816	0.00051028	0.00049952	0.00062194	0.00071217
Q5PQM9	Slc25a30	7.843e-05	0.00012431	5.68e-05	0.00020343	0.00012836	0.00010644
Q5PQN9	Mrpl38	0.00052286	0.000436	0.00052863	0.00052407	0.00046584	0.00080064
Q5PQQ1	Gtpbp3	9.206e-05	7.408e-05	2.853e-05	9.046e-05	6.444e-05	7.396e-05
Q5RK00	Mrpl46	0.00044758	0.00038643	0.00041995	0.00027962	0.00050051	0.00040791
Q5RKI8	Abcb8	0.00020974	0.00018383	9.426e-05	0.00022424	0.00014814	0.00018171
Q5RKI9	Mrrf	0.00026202	0.0002766	0.00023273	0.00025856	0.0002039	0.00029258
Q5SGE0	Lrpprc	0.00189401	0.00207284	0.00189265	0.00248528	0.00237677	0.00230981
Q5U1X1	Rexo2	0.0005816	0.00076702	0.00087283	0.00065933	0.0009836	0.00126909
Q5U2U0	Clpx	0.00065252	0.00077919	0.00117265	0.00076183	0.00068946	0.000816
Q5U2X7	Timm21	0.00013553	0.00012353	0.00012749	0.0001527	0.00012811	0.00023235
Q5XFW2	Trub2	2.794e-05	5.713e-05	5.2e-06	1.512e-05	2.116e-05	3.219e-05
Q5XHZ0	Trap1	0.00220941	0.00226313	0.00226735	0.00232328	0.00227961	0.00304903
Q5XI37	Mrps15	0.00039058	0.00037268	0.00029576	0.00030537	0.00037124	0.00041281
Q5XI79	Ndufaf7	0.00020049	0.00017204	0.00019314	0.00018821	0.00016546	0.00012341
Q5XIC2	Ecsit	0.00043794	0.00044936	0.00041151	0.00036572	0.00034484	0.00030653
Q5XIE3	Mrpl11	0.00032349	0.00030302	0.0002438	0.00048919	0.00048137	0.00044538
Q5XIE6	Hibch	0.00042193	0.0003784	0.00042313	0.00029256	0.00040345	0.00042881
Q5XIF3	Ndufs4	0.00335956	0.00359903	0.00574066	0.00440371	0.00387873	0.00313538
Q5XIH4	Lias	0.00010551	9.842e-05	7.286e-05	7.726e-05	7.062e-05	8.339e-05
Q5XII9	Mtfr1l	0.00010213	0.00013071	0.00019493	0.00023941	0.00028689	0.00017741
Q5XIJ4	Mims1	0.00047148	0.00056425	0.00051101	0.0005143	0.00058249	0.00049647
Q5XIM4	Dmac2l	0.00017566	0.00015724	0.00018128	0.00024727	0.00015721	0.00013232
Q5XIN6	Letm1	0.00359575	0.00389161	0.00335635	0.00465666	0.00447122	0.00401666
Q5XIS8	Mief1	0.00020324	9.959e-05	0.00011556	7.339e-05	0.00010976	8.644e-05
Q5XIT9	Mccc2	0.00137864	0.00158096	0.00241398	0.00141595	0.00134554	0.00146651
Q60587	Hadhb	0.0111159	0.0105692	0.00709074	0.00972334	0.0110509	0.0106269
Q62651	Ech1	0.00118467	0.00119343	0.00196052	0.0009437	0.00088549	0.00073926
Q62760	Tomm20	0.00114375	0.00200649	0.00196068	0.00270686	0.00271565	0.00252742
Q63065	Pdk1	0.00031751	0.00032983	0.00055751	0.00055498	0.00073077	0.00044807
Q63159	Coq3	4.039e-05	0.00010991	0.00013114	5.818e-05	0.00010467	9.745e-05

Accession	Gene	NOX-1	NOX-2	NOX-3	HOX-1	HOX-2	HOX-3
Q63362	Ndufa5	0.00088552	0.00105125	0.00071088	0.0008074	0.0005976	0.00066081
Q63619	Coq7	8.025e-05	0.00010693	5.904e-05	6.059e-05	5.741e-05	9.177e-05
Q63707	Dhodh	0.00026776	0.00024286	0.00020678	0.00033282	0.00032531	0.00050933
Q63750	Mrpl23	0.00023214	0.00021292	0.00011988	0.00028733	0.00029048	0.00018095
Q641X9	Mrpl9	0.00054127	0.00065342	0.00037626	0.00071858	0.00071128	0.00060137
Q641Y2	Ndufs2	0.00230998	0.00225733	0.00283501	0.00206396	0.00216585	0.00245072
Q64380	Sardh	0.00306258	0.00239017	0.00315431	0.00227255	0.00249296	0.00237819
Q64428	Hadha	0.0146023	0.0133502	0.00955796	0.00789983	0.0108377	0.0107488
Q64536	Pdk2	0.00033893	0.00028797	0.00059282	0.00029572	0.00027487	0.00027423
Q64591	Decr1	0.00229945	0.0019719	0.00297939	0.00209984	0.00189914	0.00224916
Q66H47	Mrpl24	0.00071322	0.00065507	0.00105731	0.00077978	0.00080651	0.00077989
Q66HF1	Ndufs1	0.00616741	0.006825	0.00437843	0.00656417	0.00641408	0.00580488
Q66HG6	Ca5b	9.171e-05	0.00010302	9.897e-05	5.428e-05	4.042e-05	1.701e-05
Q66HG9	Mavs	0.0006428	0.00060561	0.00037124	0.00083734	0.00077884	0.00051082
Q68FT1	Coq9	0.00027219	0.00033196	0.00072969	0.00023852	0.00032118	0.00046029
Q68FU7	Coq6	0.0238945	0.0188405	0.0197404	0.0140806	0.0211034	0.0234458
Q68FW7	Tars2	0.00052709	0.00054751	0.00066036	0.000486	0.00045326	0.00056721
Q68FX0	Idh3B	0.00365888	0.00380902	0.00299785	0.00339141	0.00383996	0.00388016
Q68FX9	Sirt5	0.00042582	0.00066946	0.00068241	0.00071126	0.00079449	0.00076675
Q68FY0	Uqcrc1	0.0090834	0.00813129	0.0140173	0.00635113	0.00710954	0.00478405
Q6AXS0	Slc8b1	0.00018234	0.00020169	7.961e-05	0.00018352	0.00019211	9.07e-05
Q6AXT0	Mrpl37	0.0007487	0.00088556	0.00065926	0.00083756	0.00080937	0.00080665
Q6AXW1	Glrx2	3.5e-05	1.187e-05	2.808e-05	5.166e-05	3.236e-05	2.72e-05
Q6AY04	Maip1	0.00037497	0.00028813	0.00021004	0.00043764	0.0003162	0.00061483
Q6AY19	Coq8b	0.00016237	0.0001538	0.0002004	0.00016057	0.00011472	0.00014725
Q6AY94	Timmdc1	5.561e-05	6.004e-05	0.00010247	0.00010795	0.00012401	9.482e-05
Q6AYL0	Slc25a19	0.00014774	0.00015192	0.00020353	0.0001578	0.00013536	0.00016404
Q6AYQ3	Fars2	5.151e-05	4.755e-05	6.378e-05	5.614e-05	3.924e-05	5.859e-05
Q6AYQ8	Fahd1	0.00048463	0.00043491	0.00085195	0.000664	0.00057799	0.0004552
Q6IM78	Top1mt	5.224e-05	3.608e-05	3.318e-05	6.563e-05	8.202e-05	5.539e-05
Q6MG21	Vars2	6.391e-05	4.372e-05	4.918e-05	5.817e-05	4.373e-05	6.437e-05
Q6P6Q6	Mterf3	6.308e-05	6.821e-05	5.502e-05	3.917e-05	3.828e-05	6.564e-05
Q6P6Q9	Micu1	0.00105205	0.0011484	0.00085034	0.00111164	0.00116367	0.0012746
Q6P6R2	Dld	0.00651601	0.00700339	0.00809254	0.00524291	0.00754426	0.00788338
Q6PCT8	Sdhd	0.00161964	0.0013312	0.00138093	0.00156163	0.00152401	0.00121334
Q6PCU8	Ndufv3	0.00049087	0.00061195	0.00068935	0.00101513	0.00087593	0.00092725
Q6PDU7	Atp5mg	0.00242187	0.00162694	0.00069578	0.00144896	0.00164548	0.00097658
Q6PDW6	Mrpl17	0.00041929	0.00030853	0.00019824	0.00030822	0.00036449	0.00039928
Q6TUF2	Sdhaf3	7.905e-05	0.00024038	0.00014642	6.154e-05	8.951e-05	8.595e-05
Q6UPE1	Etfdh	0.00424336	0.00412007	0.00338816	0.00358141	0.00364214	0.00288536
Q704E8	Abcb7	0.00039272	0.00056276	0.0002894	0.00075901	0.00047848	0.00042203
Q75Q39	Tomm70	0.00297461	0.00250208	0.00207972	0.00336222	0.00263852	0.00264579

Accession	Gene	NOX-1	NOX-2	NOX-3	HOX-1	HOX-2	HOX-3
Q75Q40	Tomm40	0.00309447	0.00223638	0.002523	0.00219504	0.00271569	0.00190672
Q75Q41	Tomm22	3.965e-05	8.485e-05	7.164e-05	0.00020776	0.00023229	7.566e-05
Q7M0E7	Mrpl14	0.0005794	0.0004917	0.00023861	0.00052811	0.00047399	0.00044529
Q7TQ16	Uqcrq	0.00203921	0.00237665	0.00168568	0.00320119	0.00369128	0.00303736
Q7TQ82	Mrps10	0.00029081	0.0003151	0.00054961	0.00047003	0.00042403	0.0003925
Q7TSA0	Rhot2	0.0004118	0.00042482	0.00029699	0.00039047	0.00039441	0.00040405
Q7TT47	Spg7	0.00045403	0.00046123	0.00059821	0.00069334	0.00057441	0.00065984
Q80W89	Ndufa11	0.00017235	0.00038562	0.00066765	0.00070233	3.968e-05	0.00027326
Q80W96	Isca1	0.00015839	0.00017194	0.00019157	0.00023739	0.00021718	0.00025053
Q811P6	Tfb1m	5.952e-05	9.37e-05	7.213e-05	0.00011832	9.726e-05	0.00011398
Q8K3P6	Slc25a25	0.00035712	0.00042189	0.00052411	0.00047786	0.00039749	0.00038477
Q8VH49	Higd1a	0.00016856	0.00010329	0.00020511	0.00036496	0.00041358	0.00047736
Q8VHF5	Cs	0.0161931	0.0151811	0.0171219	0.0182619	0.0160481	0.0218511
Q91Y94	Cox4i2	7.18e-05	9.072e-05	8.304e-05	0.00024217	0.00038384	0.00037729
Q91ZW1	Tfam	0.00088545	0.00088009	0.00090419	0.00111618	0.00105071	0.00109518
Q91ZW6	Tmlhe	0.00107521	0.00114996	0.00143611	0.00132977	0.00108621	0.00125077
Q920F5	Mlycd	0.00067873	0.00054837	0.00046007	0.00064829	0.00053487	0.00045851
Q920L2	Sdha	0.00570216	0.00513215	0.00536413	0.00553142	0.00574575	0.00591653
Q923M1	Msra	4.008e-05	5.086e-05	0.00011016	6.34e-05	5.3e-05	5.761e-05
Q924S5	Lonp1	0.00531534	0.00530259	0.00529946	0.00455486	0.00444251	0.00472647
Q99NA5	Idh3a	0.00595684	0.00766683	0.00770086	0.0112816	0.00899726	0.00846952
Q99P63	Micu2	6.253e-05	0.00016712	6.025e-05	0.00011288	0.00017574	0.00016164
Q9EPJ3	Mrps26	0.00025303	0.00037864	0.00048187	0.00038377	0.0003767	0.00084498
Q9ER34	Aco2	0.00882173	0.00894085	0.0091474	0.00891207	0.00981053	0.0116364
Q9JJW3	Atp5mk	0.00095458	0.00103276	0.00156969	0.00160233	0.00170802	0.00188515
Q9JKW1	Timm22	6.666e-05	0.00021649	0.00013457	8.997e-05	0.00016091	0.0002084
Q9JLH6	Cdk5rap1	3.43e-05	3.459e-05	4.005e-05	4.677e-05	4.134e-05	6.491e-05
Q9JM53	Aifm1	0.0029831	0.00365554	0.00351929	0.00421539	0.00336719	0.00376196
Q9NQR8	Ndufaf4	0.00049435	0.00069221	0.00045938	0.00071559	0.00100167	0.00056893
Q9QYU2	Tsfm	7.841e-05	9.133e-05	8.474e-05	7.599e-05	8.404e-05	7.944e-05
Q9R063	Prdx5	0.00180331	0.00158338	0.00249191	0.00347596	0.00364763	0.00363052
Q9WTT2	Clpb	0.00046759	0.00038485	0.00049248	0.00040213	0.00041839	0.00059169
Q9WUS0	Ak4	0.00027015	0.00031632	0.00060318	0.00067945	0.00069033	0.00068448
Q9WV97	Timm9	0.00019772	0.0001897	0.00020772	0.00029115	0.000365	0.00021483
Q9WVK7	Hadh	0.00743993	0.0053864	0.0112773	0.00406941	0.00308898	0.0058776
Q9Z0J5	Txnrd2	0.00037115	0.00037141	0.00078668	0.00041515	0.00055122	0.00099661
Q9Z0V6	Prdx3	0.00496887	0.00415881	0.00289442	0.00506485	0.00485037	0.00484041
Q9Z311	Mecr	0.00027025	0.00036081	0.00035341	0.00019138	0.00013418	0.00018333

D. Mitochondrial proteome statistics

Unpaired two-tailed Student t-test (equal variance) on log2 mitochondria-normalized intensities, hypoxia vs normoxia. FDR_BH is across all mitochondrial proteins (exploratory).

Gene	Accession	Protein	NOX_mean_log2	NOX_SD	HOX_mean_log2	HOX_SD	log2FC	FoldChange	t	p_value	FDR_BH
Tfam	Q91ZW1	Transcription factor A, mitochondrial	-10.134	0.02	-9.845	0.045	0.289	1.222	10.193	0.0005218	0.09702
Coq8a	Q5BJQ0	Atypical kinase COQ8A, mitochondrial	-11.894	0.337	-14.038	0.213	-2.144	0.226	-9.324	0.0007364	0.09702
Cox4i2	Q91Y94	Cytochrome c oxidase subunit 4 isoform 2, mitochondrial	-13.583	0.17	-11.577	0.377	2.006	4.017	8.406	0.001096	0.09702
Lonp1	Q924S5	Lon protease homolog, mitochondrial	-7.558	0.002	-7.773	0.045	-0.214	0.862	-8.246	0.00118	0.09702
Stoml2	Q4FZT0	Stomatin-like protein 2, mitochondrial	-8.289	0.082	-7.75	0.093	0.539	1.453	7.53	0.001665	0.1096
Ckmt2	P09605	Creatine kinase S-type, mitochondrial	-7.894	0.597	-10.651	0.424	-2.757	0.148	-6.522	0.002854	0.1292
Pisd	D3ZAW2	Phosphatidylserine decarboxylase proenzyme, mitochondrial	-12.371	0.072	-12.086	0.028	0.285	1.219	6.384	0.003089	0.1292
Pmpcb	Q03346	Mitochondrial-processing peptidase subunit beta	-9.407	0.063	-9.776	0.082	-0.369	0.774	-6.161	0.003524	0.1292
Hspa9	P48721	Stress-70 protein, mitochondrial	-5.632	0.069	-6.049	0.1	-0.417	0.749	-5.949	0.004005	0.1292
Cyp27a1	P17178	Sterol 26-hydroxylase, mitochondrial	-12.368	0.084	-13.195	0.236	-0.827	0.564	-5.709	0.004655	0.1292
Lrpprc	Q5SGE0	Leucine-rich PPR motif-containing protein, mitochondrial	-9.001	0.075	-8.709	0.053	0.292	1.225	5.482	0.005393	0.1292
Acss3	A0A0G2K047	Acyl-CoA synthetase short-chain family member 3, mitochondrial	-14.083	0.22	-14.886	0.127	-0.803	0.573	-5.466	0.005449	0.1292
Mrpl11	Q5XIE3	Large ribosomal subunit protein uL11m	-11.761	0.214	-11.05	0.072	0.711	1.637	5.462	0.005464	0.1292
Atp5mf	D3ZAF6	ATP synthase F(0) complex subunit f, mitochondrial	-8.897	0.268	-7.784	0.239	1.113	2.163	5.37	0.005807	0.1292
Cox6a2	P10817	Cytochrome c oxidase subunit 6A2, mitochondrial (Fragment)	-11.368	0.1	-14.582	1.036	-3.214	0.108	-5.348	0.005892	0.1292
Slc25a15	A0A0G2K309	Mitochondrial ornithine transporter 1	-12.634	0.121	-13.014	0.037	-0.379	0.769	-5.178	0.006617	0.134
Glud1	P10860	Glutamate dehydrogenase 1, mitochondrial	-6.035	0.179	-5.422	0.105	0.613	1.53	5.112	0.006925	0.134
Gls	P13264-2	Isoform 2 of Glutaminase kidney isoform, mitochondrial	-9.011	0.073	-8.329	0.227	0.682	1.604	4.953	0.007742	0.1361
Pdp1	O88483	[Pyruvate dehydrogenase [acetyl-transferring]]-phosphatase 1, mitochondrial	-11.236	0.25	-10.389	0.166	0.847	1.799	4.88	0.008162	0.1361
Eci1	P23965	Enoyl-CoA delta isomerase 1, mitochondrial	-7.856	0.137	-7.332	0.13	0.524	1.438	4.798	0.008659	0.1361
Gpd2	P35571	Glycerol-3-phosphate dehydrogenase, mitochondrial	-11.126	0.033	-10.675	0.161	0.451	1.367	4.748	0.008987	0.1361
Suox	Q07116	Sulfite oxidase, mitochondrial	-12.595	0.17	-13.425	0.254	-0.829	0.563	-4.698	0.00932	0.1361
Higd1a	Q8VH49	HIG1 domain family member 1A, mitochondrial	-12.676	0.51	-11.231	0.194	1.445	2.722	4.589	0.01011	0.1361
Prdx5	Q9R063	Peroxiredoxin-5, mitochondrial;Isoform Cytoplasmic+peroxisomal of Peroxiredoxin-5, mitochondrial	-9.022	0.337	-8.124	0.038	0.898	1.863	4.587	0.01013	0.1361
Mecr	Q9Z311	Enoyl-[acyl-carrier-protein] reductase, mitochondrial	-11.585	0.233	-12.543	0.28	-0.957	0.515	-4.56	0.01034	0.1361
Isca1	Q80W96	Iron-sulfur cluster assembly 1 homolog, mitochondrial	-12.493	0.138	-12.057	0.104	0.436	1.353	4.376	0.01191	0.1503
Uqcrq	Q7TQ16	Cytochrome b-c1 complex subunit 8	-8.956	0.248	-8.244	0.146	0.712	1.638	4.284	0.01281	0.1503
Ndufv3	Q6PCU8	NADH dehydrogenase [ubiquinone] flavoprotein 3, mitochondrial	-10.723	0.249	-10.059	0.107	0.664	1.585	4.251	0.01315	0.1503
Ecsit	Q5XIC2	Evolutionarily conserved signaling intermediate in Toll pathway, mitochondrial	-11.175	0.065	-11.53	0.13	-0.356	0.782	-4.242	0.01325	0.1503
Acadvl	P45953	Very long-chain specific acyl-CoA dehydrogenase, mitochondrial	-8.525	0.121	-9.023	0.183	-0.498	0.708	-3.935	0.01703	0.1867
Fxn	D3ZYW7	Frataxin, mitochondrial	-13.142	0.047	-12.382	0.342	0.76	1.693	3.818	0.01881	0.1996
Idh3g	P41565	Isocitrate dehydrogenase [NAD] subunit gamma 1, mitochondrial	-8.219	0.086	-8.588	0.15	-0.369	0.774	-3.69	0.02102	0.2161
Gls	P13264	Glutaminase kidney isoform, mitochondrial	-6.856	0.327	-6.179	0.061	0.677	1.599	3.524	0.02436	0.2429
Clybl	Q5I0K3	Citramalyl-CoA lyase, mitochondrial	-12.73	0.114	-13.206	0.228	-0.477	0.719	-3.245	0.03152	0.305
Slc25a11	P97700	Mitochondrial 2-oxoglutarate/malate carrier protein	-8.126	0.229	-7.677	0.088	0.449	1.366	3.177	0.03365	0.3152
Letm1	Q5XIN6	Mitochondrial proton/calcium exchanger protein	-8.115	0.107	-7.837	0.11	0.277	1.212	3.131	0.03514	0.3152
Acadm	P08503	Medium-chain specific acyl-CoA dehydrogenase, mitochondrial	-8.224	0.143	-7.825	0.17	0.399	1.319	3.11	0.03586	0.3152
Bdh1	P29147	D-beta-hydroxybutyrate dehydrogenase, mitochondrial	-9.001	0.093	-8.226	0.427	0.775	1.711	3.073	0.03718	0.3152

Gene	Accession	Protein	NOX_mean_log2	NOX_SD	HOX_mean_log2	HOX_SD	log2FC	FoldChange	t	p_value	FDR_BH
Ca5b	Q66HG6	Carbonic anhydrase 5B, mitochondrial	-13.32	0.085	-14.869	0.87	-1.549	0.342	-3.068	0.03736	0.3152
Ivd	P12007	Isovaleryl-CoA dehydrogenase, mitochondrial	-8.221	0.305	-8.761	0.06	-0.539	0.688	-3.008	0.03962	0.3259
Top1mt	Q6IM78	DNA topoisomerase I, mitochondrial	-14.621	0.348	-13.87	0.284	0.751	1.683	2.894	0.04438	0.3562
Dhodh	Q63707	Dihydroorotate dehydrogenase (quinone), mitochondrial	-12.038	0.188	-11.359	0.364	0.679	1.601	2.866	0.04563	0.3574
Hspd1	P63039	60 kDa heat shock protein, mitochondrial	-4.829	0.148	-5.384	0.309	-0.555	0.681	-2.802	0.04871	0.3715
Atp5pf	P21571	ATP synthase peripheral stalk subunit F6, mitochondrial	-9.646	0.224	-8.981	0.35	0.665	1.585	2.77	0.05032	0.3715
Ech1	Q62651	Delta(3,5)-Delta(2,4)-dienoyl-CoA isomerase, mitochondrial	-9.476	0.417	-10.197	0.183	-0.722	0.606	-2.749	0.05143	0.3715
Tfb1m	Q811P6	Dimethyladenosine transferase 1, mitochondrial	-13.726	0.329	-13.157	0.15	0.568	1.483	2.725	0.0527	0.3715
Mt-cyb	P00159	Cytochrome b	-11.302	0.522	-10.467	0.105	0.835	1.784	2.718	0.05307	0.3715
Timm10	P62074	Mitochondrial import inner membrane translocase subunit Tim10	-14.116	0.605	-13.011	0.428	1.105	2.151	2.583	0.06113	0.3916
Gfm1	Q07803	Elongation factor G, mitochondrial	-10.841	0.096	-10.561	0.161	0.279	1.214	2.58	0.0613	0.3916
Suclg1	P13086	Succinate--CoA ligase [ADP/GDP-forming] subunit alpha, mitochondrial	-8.019	0.36	-7.326	0.295	0.692	1.616	2.575	0.06166	0.3916
Uqcrc1	Q68FY0	Cytochrome b-c1 complex subunit 1, mitochondrial	-6.627	0.415	-7.381	0.294	-0.754	0.593	-2.564	0.06236	0.3916
Mtarc2	O88994	Mitochondrial amidoxime reducing component 2	-9.216	0.224	-8.83	0.134	0.386	1.307	2.557	0.06282	0.3916
Tomm20	Q62760	Mitochondrial import receptor subunit TOM20 homolog	-9.243	0.459	-8.561	0.059	0.682	1.604	2.553	0.06308	0.3916
Atp5mk	Q9JJW3	ATP synthase F(0) complex subunit k, mitochondrial	-9.756	0.386	-9.177	0.118	0.579	1.494	2.486	0.06774	0.4076
Ak4	Q9WUS0	Adenylate kinase 4, mitochondrial	-11.392	0.614	-10.512	0.011	0.88	1.84	2.481	0.06813	0.4076
Idh3a	Q99NA5	Isocitrate dehydrogenase [NAD] subunit alpha, mitochondrial	-7.146	0.212	-6.717	0.218	0.43	1.347	2.448	0.07062	0.4149
Cox5a	P11240	Cytochrome c oxidase subunit 5A, mitochondrial	-7.436	0.428	-6.727	0.277	0.709	1.634	2.407	0.07378	0.4259
Ttc19	D4A6D7	Tetratricopeptide repeat protein 19, mitochondrial	-14.202	0.209	-13.352	0.581	0.851	1.803	2.387	0.07541	0.4278
Aldh5a1	P51650	Succinate-semialdehyde dehydrogenase, mitochondrial;Isoform Short of Succinate-semialdehyde dehydrogenase, mitochondrial	-13.579	0.719	-14.96	0.721	-1.381	0.384	-2.349	0.07856	0.4299
Spg7	Q7TT47	Mitochondrial inner membrane m-AAA protease component paraplegin;Isoform 2 of Mitochondrial inner membrane m-AAA protease component paraplegin	-10.965	0.223	-10.608	0.141	0.356	1.28	2.337	0.07965	0.4299
Slc25a1	P32089	Tricarboxylate transport protein, mitochondrial	-7.821	0.36	-7.289	0.175	0.532	1.446	2.301	0.08287	0.4299
Timm9	Q9WV97	Mitochondrial import inner membrane translocase subunit Tim9	-12.3	0.066	-11.783	0.384	0.517	1.431	2.3	0.08291	0.4299
Ccdc90b	Q4V897	Coiled-coil domain-containing protein 90B, mitochondrial	-11.256	0.144	-11.058	0.041	0.198	1.147	2.281	0.08466	0.4299
Gcsh	Q5I0P2	Glycine cleavage system H protein, mitochondrial	-11.074	0.302	-10.441	0.374	0.633	1.551	2.28	0.08481	0.4299
Atp5pb	P19511	ATP synthase peripheral stalk subunit b, mitochondrial	-6.903	0.299	-6.36	0.284	0.543	1.457	2.278	0.08493	0.4299
Cdk5rap1	Q9JLH6	Mitochondrial tRNA methylthiotransferase CDK5RAP1	-14.753	0.126	-14.286	0.336	0.467	1.382	2.252	0.08748	0.4361
Mtfr1l	Q5XII9	Mitochondrial fission regulator 1-like	-12.828	0.471	-12.085	0.35	0.742	1.673	2.192	0.09347	0.4512
Mrps7	Q5I0K8	Small ribosomal subunit protein uS7m	-11.569	0.178	-11.291	0.134	0.277	1.212	2.154	0.0975	0.4512
Tomm22	Q75Q41	Mitochondrial import receptor subunit TOM22 homolog	-13.972	0.576	-12.665	0.892	1.307	2.474	2.133	0.09991	0.4512
Gpam	P97564	Glycerol-3-phosphate acyltransferase 1, mitochondrial	-13.437	0.165	-12.885	0.418	0.553	1.467	2.131	0.1001	0.4512
Hadh	Q9WVK7	Hydroxyacyl-coenzyme A dehydrogenase, mitochondrial	-7.026	0.534	-7.897	0.466	-0.871	0.547	-2.128	0.1004	0.4512
Pccb	P07633	Propionyl-CoA carboxylase beta chain, mitochondrial	-10.135	0.139	-10.327	0.073	-0.192	0.875	-2.113	0.1021	0.4512
Timmdc1	Q6AY94	Complex I assembly factor TIMMDC1, mitochondrial	-13.804	0.48	-13.173	0.194	0.63	1.548	2.109	0.1026	0.4512
Mpc1	P63031	Mitochondrial pyruvate carrier 1	-10.145	0.415	-9.588	0.199	0.558	1.472	2.099	0.1037	0.4512
Uqcrfs1	P20788	Cytochrome b-c1 complex subunit Rieske, mitochondrial	-6.762	0.214	-7.059	0.119	-0.297	0.814	-2.098	0.1039	0.4512
Ccdc51	Q5PPN7	Mitochondrial potassium channel	-11.038	0.125	-10.691	0.258	0.347	1.272	2.095	0.1042	0.4512
Atp5f1d	P35434	ATP synthase F(1) complex subunit delta, mitochondrial	-7.32	0.161	-6.836	0.374	0.484	1.399	2.062	0.1082	0.4623
Akap1	O88884	A-kinase anchor protein 1, mitochondrial	-12.075	0.265	-11.758	0.042	0.316	1.245	2.039	0.1111	0.4686

Gene	Accession	Protein	NOX_mean_log2	NOX_SD	HOX_mean_log2	HOX_SD	log2FC	FoldChange	t	p_value	FDR_BH
Qrsl1	Q5FWT5	Glutamyl-tRNA(Gln) amidotransferase subunit A, mitochondrial	-14.55	0.277	-15.157	0.441	-0.606	0.657	-2.017	0.1139	0.4744
Ak2	P29410	Adenylate kinase 2, mitochondrial;Isoform 2 of Adenylate kinase 2, mitochondrial	-9.249	0.29	-8.6	0.485	0.648	1.567	1.987	0.1179	0.4808
Tmem11	B0BN86	Transmembrane protein 11, mitochondrial	-9.804	0.377	-9.369	0.037	0.434	1.351	1.983	0.1184	0.4808
Sardh	Q64380	Sarcosine dehydrogenase, mitochondrial	-8.456	0.22	-8.715	0.067	-0.259	0.836	-1.953	0.1225	0.4916
Acaa2	P13437	3-ketoacyl-CoA thiolase, mitochondrial	-8.723	0.51	-9.346	0.255	-0.623	0.65	-1.892	0.1314	0.5209
Coq10b	Q5I0I9	Coenzyme Q-binding protein COQ10 homolog B, mitochondrial	-14.128	0.39	-13.692	0.103	0.436	1.353	1.873	0.1344	0.5265
Cps1	P07756	Carbamoyl-phosphate synthase [ammonia], mitochondrial	-10.955	0.891	-11.975	0.327	-1.02	0.493	-1.862	0.1361	0.5267
Slc25a30	Q5PQM9	Kidney mitochondrial carrier protein 1	-13.572	0.568	-12.796	0.481	0.776	1.712	1.806	0.1453	0.5558
Atp5f1b	P10719	ATP synthase F(1) complex catalytic subunit beta, mitochondrial	-4.431	0.232	-4.754	0.21	-0.323	0.799	-1.791	0.1477	0.5577
Aldh4a1	P0C2X9	Delta-1-pyrroline-5-carboxylate dehydrogenase, mitochondrial	-9.816	0.571	-10.446	0.218	-0.63	0.646	-1.783	0.1492	0.5577
Atp5f1e	P29418	ATP synthase F(1) complex subunit epsilon, mitochondrial	-11.155	2.061	-8.802	1.143	2.353	5.107	1.729	0.1589	0.5661
Maip1	Q6AY04	m-AAA protease-interacting protein 1, mitochondrial	-11.786	0.419	-11.151	0.48	0.636	1.554	1.729	0.1589	0.5661
Sdhaf3	Q6TUF2	Succinate dehydrogenase assembly factor 3, mitochondrial	-12.796	0.804	-13.647	0.297	-0.852	0.554	-1.722	0.1602	0.5661
Aass	A2VCW9	Alpha-aminoadipic semialdehyde synthase, mitochondrial	-13.195	0.246	-13.621	0.355	-0.426	0.744	-1.708	0.1629	0.5661
Mrpl16	Q5M818	Large ribosomal subunit protein uL16m	-11.91	0.176	-11.476	0.406	0.434	1.351	1.699	0.1645	0.5661
Sirt5	Q68FX9	NAD-dependent protein deacylase sirtuin-5, mitochondrial	-10.753	0.385	-10.368	0.082	0.385	1.306	1.694	0.1654	0.5661
Ndufa5	Q63362	NADH dehydrogenase [ubiquinone] 1 alpha subcomplex subunit 5	-10.164	0.283	-10.515	0.221	-0.351	0.784	-1.694	0.1655	0.5661
Sdha	Q920L2	Succinate dehydrogenase [ubiquinone] flavoprotein subunit, mitochondrial	-7.534	0.076	-7.447	0.049	0.087	1.062	1.662	0.1719	0.5661
Rpusd4	Q4QQT0	Pseudouridylate synthase RPUSD4, mitochondrial	-14.226	0.395	-13.822	0.156	0.404	1.323	1.647	0.175	0.5661
Mccc1	Q5I0C3	Methylcrotonoyl-CoA carboxylase subunit alpha, mitochondrial	-10.145	0.196	-10.42	0.213	-0.275	0.827	-1.646	0.1752	0.5661
Pdk1	Q63065	[Pyruvate dehydrogenase (acetyl-transferring)] kinase isozyme 1, mitochondrial	-11.332	0.454	-10.786	0.354	0.546	1.46	1.643	0.1756	0.5661
Mief1	Q5XIS8	Mitochondrial dynamics protein MID51	-12.879	0.543	-13.462	0.292	-0.583	0.668	-1.637	0.177	0.5661
Ndufa9	Q5BK63	NADH dehydrogenase [ubiquinone] 1 alpha subcomplex subunit 9, mitochondrial	-8.912	0.109	-9.056	0.108	-0.145	0.904	-1.629	0.1786	0.5661
Etfa	P13803	Electron transfer flavoprotein subunit alpha, mitochondrial	-7.611	0.086	-7.344	0.271	0.267	1.203	1.625	0.1794	0.5661
Mrpl9	Q641X9	Large ribosomal subunit protein bL9m	-10.936	0.405	-10.533	0.144	0.403	1.322	1.623	0.18	0.5661
Mrpl22	P0C2C0	Large ribosomal subunit protein uL22m	-11.05	0.274	-11.537	0.444	-0.486	0.714	-1.613	0.182	0.5661
Acadl	P15650	Long-chain specific acyl-CoA dehydrogenase, mitochondrial	-7.733	0.13	-7.441	0.285	0.292	1.224	1.613	0.1821	0.5661
Micu1	Q6P6Q9	Calcium uptake protein 1, mitochondrial	-9.953	0.223	-9.725	0.1	0.227	1.171	1.611	0.1824	0.5661
Tomm40l	A4F267	Mitochondrial import receptor subunit TOM40B	-11.537	0.813	-10.791	0.059	0.746	1.677	1.584	0.1884	0.5707
Rhot1	A1L1L6	Mitochondrial Rho GTPase	-10.484	0.489	-9.959	0.303	0.525	1.439	1.582	0.1888	0.5707
Coq8b	Q6AY19	Atypical kinase COQ8B, mitochondrial	-12.513	0.202	-12.808	0.252	-0.295	0.815	-1.581	0.1891	0.5707
Mterf3	Q6P6Q6	Transcription termination factor 3, mitochondrial	-13.981	0.157	-14.403	0.44	-0.422	0.746	-1.565	0.1925	0.5759
Tefm	Q4KM51	Transcription elongation factor, mitochondrial	-13.412	0.382	-13.802	0.214	-0.39	0.763	-1.543	0.1977	0.5781
Mt-co3	P05505	Cytochrome c oxidase subunit 3	-8.3	0.409	-8.959	0.622	-0.659	0.633	-1.534	0.1998	0.5781
Ndufaf4	Q9NQR8	NADH dehydrogenase [ubiquinone] 1 alpha subcomplex assembly factor 4	-10.856	0.315	-10.397	0.41	0.458	1.374	1.534	0.1998	0.5781
Cox7a2	P35171	Cytochrome c oxidase subunit 7A2, mitochondrial	-10.355	0.567	-9.818	0.217	0.537	1.451	1.532	0.2003	0.5781
Mrpl42	P0C2B9	Large ribosomal subunit protein mL42	-13.125	0.058	-12.735	0.439	0.39	1.31	1.522	0.2026	0.5795
Cox5b	P12075	Cytochrome c oxidase subunit 5B, mitochondrial	-6.891	0.411	-6.525	0.09	0.367	1.289	1.511	0.2053	0.5815
Micu2	Q99P63	Calcium uptake protein 2, mitochondrial	-13.51	0.835	-12.727	0.339	0.783	1.72	1.505	0.2068	0.5815
Aars2	D3ZX08	Alanine--tRNA ligase, mitochondrial	-11.906	0.239	-12.119	0.067	-0.213	0.862	-1.486	0.2114	0.5837
Etfdh	Q6UPE1	Electron transfer flavoprotein-ubiquinone oxidoreductase, mitochondrial	-8.003	0.176	-8.221	0.187	-0.218	0.86	-1.468	0.2161	0.5837
Tars2	Q68FW7	Threonine--tRNA ligase, mitochondrial	-10.763	0.174	-10.966	0.166	-0.203	0.869	-1.463	0.2172	0.5837

Gene	Accession	Protein	NOX_mean_log2	NOX_SD	HOX_mean_log2	HOX_SD	log2FC	FoldChange	t	p_value	FDR_BH
Cs	Q8VHF5	Citrate synthase, mitochondrial	-5.953	0.087	-5.751	0.224	0.202	1.15	1.457	0.2188	0.5837
Aco2	Q9ER34	Aconitate hydratase, mitochondrial	-6.801	0.026	-6.636	0.195	0.165	1.121	1.455	0.2192	0.5837
Pdk2	Q64536	[Pyruvate dehydrogenase (acetyl-transferring)] kinase isozyme 2, mitochondrial	-11.336	0.546	-11.795	0.062	-0.459	0.728	-1.445	0.222	0.5837
Pgam5	Q562B5	Serine/threonine-protein phosphatase PGAM5, mitochondrial	-8.915	0.392	-8.587	0.037	0.328	1.255	1.442	0.2227	0.5837
Acot2	O55171	Acyl-coenzyme A thioesterase 2, mitochondrial	-9.189	0.242	-9.49	0.271	-0.301	0.812	-1.436	0.2243	0.5837
Prdx3	Q9Z0V6	Thioredoxin-dependent peroxide reductase, mitochondrial	-7.998	0.397	-7.668	0.037	0.33	1.257	1.434	0.2248	0.5837
Prorp	B5DF07	Mitochondrial ribonuclease P catalytic subunit	-14.636	0.474	-14.245	0.016	0.39	1.311	1.426	0.227	0.5837
Cox6a1	P10818	Cytochrome c oxidase subunit 6A1, mitochondrial	-12.905	1.651	-11.061	1.515	1.844	3.59	1.425	0.2273	0.5837
Hadha	Q64428	Trifunctional enzyme subunit alpha, mitochondrial	-6.345	0.322	-6.684	0.26	-0.339	0.79	-1.419	0.2289	0.5837
Timm21	Q5U2X7	Mitochondrial import inner membrane translocase subunit Tim21	-12.923	0.068	-12.56	0.441	0.364	1.287	1.41	0.2313	0.5855
Nme6	O88426	Nucleoside diphosphate kinase 6, mitochondrial	-13.049	0.241	-12.597	0.51	0.453	1.369	1.389	0.2371	0.5925
Atp5pd	P31399	ATP synthase peripheral stalk subunit d, mitochondrial	-7.102	0.476	-6.666	0.268	0.436	1.353	1.383	0.2388	0.5925
Ndufaf7	Q5XI79	Protein arginine methyltransferase NDUFAF7, mitochondrial	-12.376	0.115	-12.64	0.312	-0.265	0.832	-1.378	0.2404	0.5925
Lias	Q5XIH4	Lipoyl synthase, mitochondrial	-13.422	0.284	-13.666	0.12	-0.245	0.844	-1.374	0.2413	0.5925
Dlat	P08461	Dihydrolipoyllysine-residue acetyltransferase component of pyruvate dehydrogenase complex, mitochondrial	-6.869	0.208	-7.088	0.186	-0.219	0.859	-1.359	0.2456	0.5985
Sod2	P07895	Superoxide dismutase [Mn], mitochondrial	-7.676	0.389	-7.335	0.2	0.341	1.267	1.35	0.2483	0.6007
Tufm	P85834	Elongation factor Tu, mitochondrial	-6.962	0.173	-7.123	0.114	-0.161	0.895	-1.342	0.2508	0.6023
Slc25a20	P97521	Mitochondrial carnitine/acylcarnitine carrier protein	-7.294	0.182	-7.47	0.14	-0.176	0.885	-1.328	0.2549	0.6077
Bckdhb	P35738	2-oxoisovalerate dehydrogenase subunit beta, mitochondrial	-9.214	0.957	-9.978	0.329	-0.764	0.589	-1.307	0.2611	0.6149
Cpox	Q3B7D0	Oxygen-dependent coproporphyrinogen-III oxidase, mitochondrial	-11.353	0.554	-10.922	0.143	0.432	1.349	1.306	0.2617	0.6149
Sdhb	P21913	Succinate dehydrogenase [ubiquinone] iron-sulfur subunit, mitochondrial	-8.154	0.199	-7.893	0.286	0.261	1.198	1.3	0.2635	0.6149
Eral1	Q5EBA0	GTPase Era, mitochondrial;Isoform 2 of GTPase Era, mitochondrial	-14.566	0.096	-14.904	0.444	-0.338	0.791	-1.287	0.2675	0.6197
Cox4i1	P10888	Cytochrome c oxidase subunit 4 isoform 1, mitochondrial	-5.82	0.158	-5.949	0.082	-0.13	0.914	-1.262	0.2755	0.6338
Trap1	Q5XHZ0	Heat shock protein 75 kDa, mitochondrial	-8.798	0.021	-8.628	0.235	0.17	1.125	1.25	0.2794	0.6339
Mrpl30	P0C2C1	Large ribosomal subunit protein uL30m	-11.765	0.457	-11.398	0.224	0.367	1.29	1.25	0.2795	0.6339
Ndufaf3	O08776	NADH dehydrogenase [ubiquinone] 1 alpha subcomplex assembly factor 3;Isoform 4 of NADH dehydrogenase [ubiquinone] 1 alpha subcomplex assembly factor 3	-11.519	0.555	-12.156	0.695	-0.637	0.643	-1.24	0.2827	0.6339
Mccc2	Q5XIT9	Methylcrotonoyl-CoA carboxylase beta chain, mitochondrial	-9.167	0.421	-9.472	0.062	-0.304	0.81	-1.238	0.2835	0.6339
Hspe1	P26772	10 kDa heat shock protein, mitochondrial	-4.668	0.201	-4.966	0.368	-0.298	0.813	-1.233	0.2851	0.6339
Aifm1	Q9JM53	Apoptosis-inducing factor 1, mitochondrial	-8.212	0.156	-8.053	0.162	0.159	1.116	1.223	0.2884	0.6367
Mrpl23	Q63750	Large ribosomal subunit protein uL23m	-12.432	0.518	-11.982	0.39	0.45	1.366	1.202	0.2957	0.6472
Mavs	Q66HG9	Mitochondrial antiviral-signaling protein	-10.896	0.435	-10.494	0.385	0.402	1.321	1.198	0.297	0.6472
Glrx2	Q6AXW1	Glutaredoxin-2, mitochondrial;Isoform 2 of Glutaredoxin-2, mitochondrial	-15.428	0.825	-14.774	0.478	0.654	1.574	1.189	0.3003	0.6501
Pdp2	O88484	[Pyruvate dehydrogenase [acetyl-transferring]]-phosphatase 2, mitochondrial	-13.662	0.438	-13.356	0.106	0.305	1.236	1.175	0.3051	0.6562
Grpel1	P97576	GrpE protein homolog 1, mitochondrial	-8.259	0.106	-8.094	0.224	0.165	1.122	1.155	0.3125	0.6648
Hmgcs2	P22791	Hydroxymethylglutaryl-CoA synthase, mitochondrial	-13.397	1.009	-14.092	0.267	-0.694	0.618	-1.153	0.3132	0.6648
Hibadh	P29266	3-hydroxyisobutyrate dehydrogenase, mitochondrial	-8.609	0.106	-8.749	0.183	-0.14	0.908	-1.145	0.316	0.6664
Mt-co2	P00406	Cytochrome c oxidase subunit 2	-6.308	0.575	-7.027	0.93	-0.719	0.608	-1.138	0.3185	0.6675
Tsfm	Q9QYU2	Elongation factor Ts, mitochondrial	-13.528	0.11	-13.614	0.073	-0.086	0.942	-1.13	0.3218	0.6682
Ndufs2	Q641Y2	NADH dehydrogenase [ubiquinone] iron-sulfur protein 2, mitochondrial	-8.67	0.181	-8.815	0.128	-0.144	0.905	-1.127	0.3229	0.6682
Abcb7	Q704E8	Iron-sulfur clusters transporter ABCB7, mitochondrial	-11.288	0.48	-10.868	0.446	0.42	1.338	1.111	0.3289	0.6739

Gene	Accession	Protein	NOX_mean_log2	NOX_SD	HOX_mean_log2	HOX_SD	log2FC	FoldChange	t	p_value	FDR_BH
Rexo2	Q5U1X1	Oligoribonuclease, mitochondrial	-10.419	0.299	-10.059	0.476	0.36	1.283	1.109	0.3298	0.6739
Uqcrc2	P32551	Cytochrome b-c1 complex subunit 2, mitochondrial	-6.741	0.234	-6.899	0.09	-0.158	0.896	-1.091	0.3364	0.6764
Msrb2	Q4FZX5	Methionine-R-sulfoxide reductase B2, mitochondrial	-12.274	0.371	-12.015	0.178	0.259	1.197	1.09	0.3368	0.6764
Acad9	B1WC61	Complex I assembly factor ACAD9, mitochondrial	-8.441	0.118	-8.31	0.172	0.131	1.095	1.089	0.3372	0.6764
Mrps31	B0BN56	Small ribosomal subunit protein mS31	-10.756	0.219	-10.614	0.055	0.141	1.103	1.082	0.34	0.6779
Tomm70	Q75Q39	Mitochondrial import receptor subunit TOM70	-8.648	0.258	-8.448	0.201	0.2	1.149	1.06	0.3488	0.6913
Decr1	Q64591	2,4-dienoyl-CoA reductase [(3E)-enoyl-CoA-producing], mitochondrial	-8.714	0.301	-8.911	0.123	-0.197	0.872	-1.05	0.3531	0.6953
Ak3	P29411	GTP:AMP phosphotransferase AK3, mitochondrial	-7.034	0.34	-6.778	0.252	0.255	1.194	1.045	0.355	0.6953
Atp5mc1	Q06645	ATP synthase F(0) complex subunit C1, mitochondrial;ATP synthase F(0) complex subunit C2, mitochondrial;ATP synthase F(0) complex subunit C3, mitochondrial	-10.047	0.189	-9.728	0.505	0.319	1.247	1.024	0.3638	0.7031
Tomm40	Q75Q40	Mitochondrial import receptor subunit TOM40 homolog	-8.59	0.237	-8.797	0.257	-0.206	0.867	-1.023	0.364	0.7031
Slc25a12	F1LX07	Electrogenic aspartate/glutamate antiporter SLC25A12, mitochondrial	-8.999	0.182	-9.208	0.304	-0.209	0.865	-1.02	0.3654	0.7031
Slc25a10	O89035	Mitochondrial dicarboxylate carrier	-10.309	0.218	-10.572	0.395	-0.263	0.833	-1.009	0.3702	0.7081
Mrps26	Q9EPJ3	Small ribosomal subunit protein mS26	-11.445	0.47	-10.977	0.665	0.468	1.383	0.995	0.3759	0.7129
Coq4	Q4FZU1	Ubiquinone biosynthesis protein COQ4 homolog, mitochondrial	-13.246	0.296	-13.55	0.439	-0.304	0.81	-0.993	0.377	0.7129
Mrpl10	P0C2C4	Large ribosomal subunit protein uL10m	-12.447	0.517	-12.775	0.296	-0.328	0.797	-0.953	0.3946	0.7419
Tcaim	P0DKR2	T-cell activation inhibitor, mitochondrial	-14.72	0.214	-14.531	0.275	0.19	1.141	0.944	0.3987	0.7446
Echs1	P14604	Enoyl-CoA hydratase, mitochondrial	-8.275	0.184	-8.402	0.146	-0.127	0.916	-0.938	0.4013	0.7446
Pcca	P14882	Propionyl-CoA carboxylase alpha chain, mitochondrial	-9.856	0.102	-9.755	0.157	0.101	1.072	0.93	0.4052	0.7446
Ndufaf5	B2GV71	Arginine-hydroxylase NDUFAF5, mitochondrial	-12.879	0.644	-12.505	0.279	0.373	1.295	0.921	0.409	0.7446
Hagh	O35952	Hydroxyacylglutathione hydrolase, mitochondrial	-11.123	0.239	-10.894	0.358	0.229	1.172	0.92	0.4096	0.7446
Mrpl38	Q5PQN9	Large ribosomal subunit protein mL38	-10.983	0.156	-10.751	0.411	0.233	1.175	0.916	0.4113	0.7446
Mt-nd2	P11662	NADH-ubiquinone oxidoreductase chain 2	-15.008	2.666	-12.907	2.981	2.102	4.292	0.91	0.4141	0.7446
Got2	P00507	Aspartate aminotransferase, mitochondrial	-6.529	0.395	-6.311	0.126	0.218	1.163	0.91	0.4142	0.7446
Slc25a16	P16261	Mitochondrial coenzyme A transporter SLC25A16	-12.794	0.166	-12.59	0.365	0.204	1.152	0.883	0.427	0.7635
Mrpl37	Q6AXT0	Large ribosomal subunit protein mL37	-10.364	0.214	-10.256	0.03	0.108	1.078	0.865	0.4358	0.775
Mt-atp6	P05504	ATP synthase F(0) complex subunit a	-12.503	0.511	-12.243	0.168	0.261	1.198	0.839	0.4485	0.7934
Nadk2	Q1HCL7	NAD kinase 2, mitochondrial	-12.974	0.238	-12.784	0.32	0.189	1.14	0.822	0.4574	0.8048
Mrpl17	Q6PDW6	Large ribosomal subunit protein bL17m	-11.728	0.543	-11.459	0.189	0.269	1.205	0.809	0.4637	0.8114
Pdha1	P26284	Pyruvate dehydrogenase E1 component subunit alpha, somatic form, mitochondrial	-7.534	0.311	-7.382	0.109	0.152	1.111	0.797	0.4699	0.8179
Hibch	Q5XIE6	3-hydroxyisobutyryl-CoA hydrolase, mitochondrial	-11.262	0.092	-11.401	0.296	-0.139	0.908	-0.775	0.4816	0.8338
Abhd10	Q5I0K5	Palmitoyl-protein thioesterase ABHD10, mitochondrial	-12.499	1.246	-11.946	0.228	0.552	1.467	0.755	0.4921	0.8366
Slc25a19	Q6AYL0	Mitochondrial thiamine pyrophosphate carrier	-12.557	0.256	-12.685	0.147	-0.128	0.915	-0.749	0.4957	0.8366
Idh3B	Q68FX0	Isocitrate dehydrogenase [NAD] subunit beta, mitochondrial	-8.171	0.185	-8.079	0.108	0.091	1.065	0.739	0.5008	0.8366
Mthfd2l	D3ZUA0	Bifunctional methylenetetrahydrofolate dehydrogenase/cyclohydrolase 2, mitochondrial	-13.545	1.356	-12.956	0.288	0.588	1.504	0.735	0.503	0.8366
Atad1	Q505J9	Outer mitochondrial transmembrane helix translocase	-9.775	0.283	-9.904	0.121	-0.129	0.914	-0.728	0.5071	0.8366
Mt-nd1	P03889	NADH-ubiquinone oxidoreductase chain 1	-10.099	0.626	-10.367	0.136	-0.268	0.831	-0.724	0.5091	0.8366
Slc25a13	F1LZW6	Electrogenic aspartate/glutamate antiporter SLC25A13, mitochondrial	-9.091	0.14	-9.176	0.152	-0.085	0.943	-0.716	0.5135	0.8366
Dars2	Q3KRD0	Aspartate--tRNA ligase, mitochondrial	-12.483	0.3	-12.642	0.24	-0.158	0.896	-0.715	0.5143	0.8366
Hadhb	Q60587	Trifunctional enzyme subunit beta, mitochondrial	-6.732	0.355	-6.58	0.095	0.152	1.111	0.714	0.5145	0.8366
Gtpbp3	Q5PQQ1	5-taurinomethyluridine-[tRNA] synthase subunit GTPB3, mitochondrial	-14.075	0.899	-13.692	0.246	0.383	1.304	0.711	0.5164	0.8366
Mrps10	Q7TQ82	Small ribosomal subunit protein uS10m	-11.403	0.5	-11.191	0.13	0.212	1.158	0.71	0.5171	0.8366

Gene	Accession	Protein	NOX_mean_log2	NOX_SD	HOX_mean_log2	HOX_SD	log2FC	FoldChange	t	p_value	FDR_BH
Slc25a4	Q05962	ADP/ATP translocase 1	-3.471	0.22	-3.566	0.076	-0.095	0.936	-0.708	0.5179	0.8366
Atp5me	P29419	ATP synthase F(0) complex subunit e, mitochondrial	-8.489	0.371	-8.674	0.272	-0.185	0.879	-0.698	0.5236	0.8366
Ssbp1	P28042	Single-stranded DNA-binding protein, mitochondrial	-8.844	0.344	-8.574	0.575	0.27	1.206	0.697	0.5244	0.8366
Ppif	P29117	Peptidyl-prolyl cis-trans isomerase F, mitochondrial	-10.698	0.285	-10.824	0.131	-0.126	0.916	-0.695	0.5252	0.8366
Uqcrh	Q5M9I5	Cytochrome b-c1 complex subunit 6, mitochondrial	-11.912	0.475	-11.686	0.304	0.226	1.169	0.693	0.5267	0.8366
Txnrd2	Q9Z0J5	Thioredoxin reductase 2, mitochondrial;Isoform 2 of Thioredoxin reductase 2, mitochondrial	-11.034	0.625	-10.677	0.645	0.357	1.281	0.689	0.5285	0.8366
Fdx1	P24483	Adrenodoxin, mitochondrial	-11.046	0.446	-10.863	0.132	0.183	1.135	0.681	0.533	0.8366
Slc25a38	Q499U1	Mitochondrial glycine transporter	-15.316	1.112	-15.81	0.588	-0.494	0.71	-0.68	0.5338	0.8366
Coq7	Q63619	NADPH-dependent 3-demethoxyubiquinone 3-hydroxylase, mitochondrial	-13.615	0.429	-13.837	0.37	-0.222	0.857	-0.68	0.5341	0.8366
Ndufs1	Q66HF1	NADH-ubiquinone oxidoreductase 75 kDa subunit, mitochondrial	-7.457	0.336	-7.321	0.094	0.136	1.099	0.674	0.537	0.8366
Pc	P52873	Pyruvate carboxylase, mitochondrial	-8.701	0.07	-8.733	0.046	-0.032	0.978	-0.671	0.5391	0.8366
Mdh2	P04636	Malate dehydrogenase, mitochondrial	-5.324	0.018	-5.363	0.102	-0.038	0.974	-0.642	0.5557	0.85
Fdxr	P56522	NADPH:adrenodoxin oxidoreductase, mitochondrial	-10.929	0.234	-11.038	0.183	-0.109	0.927	-0.634	0.5607	0.85
Ndufa11	Q80W89	NADH dehydrogenase [ubiquinone] 1 alpha subcomplex subunit 11	-11.464	0.983	-12.311	2.113	-0.848	0.556	-0.63	0.5629	0.85
Sdhaf1	B0K036	Succinate dehydrogenase assembly factor 1, mitochondrial	-11.908	0.552	-12.304	0.951	-0.396	0.76	-0.624	0.5666	0.85
Abcb8	Q5RKI8	Mitochondrial potassium channel ATP-binding subunit	-12.667	0.619	-12.423	0.299	0.244	1.184	0.615	0.5718	0.85
Slc25a22	A0A0G2K5L2	Mitochondrial glutamate carrier 1	-10.083	0.263	-9.93	0.342	0.152	1.111	0.612	0.5735	0.85
Oat	P04182	Ornithine aminotransferase, mitochondrial	-7.241	0.3	-7.107	0.231	0.134	1.097	0.612	0.5735	0.85
Clpx	Q5U2U0	ATP-dependent clpX-like chaperone, mitochondrial	-10.214	0.434	-10.373	0.122	-0.159	0.896	-0.61	0.5747	0.85
Cpt2	P18886	Carnitine O-palmitoyltransferase 2, mitochondrial	-9.24	0.121	-9.304	0.137	-0.064	0.957	-0.606	0.5771	0.85
Slc25a51	Q52KK3	Mitochondrial nicotinamide adenine dinucleotide transporter SLC25A51	-11.424	0.444	-11.265	0.103	0.159	1.117	0.605	0.5779	0.85
Mtfmt	Q5I0C5	Methionyl-tRNA formyltransferase, mitochondrial	-14.815	1.186	-14.401	0.15	0.414	1.332	0.6	0.5809	0.85
D2hgdh	P84850	D-2-hydroxyglutarate dehydrogenase, mitochondrial	-12.045	0.236	-12.156	0.217	-0.111	0.926	-0.598	0.5819	0.85
Pars2	Q5M7W7	Probable proline--tRNA ligase, mitochondrial	-12.696	0.077	-12.843	0.421	-0.147	0.903	-0.595	0.5838	0.85
Mrpl14	Q7M0E7	Large ribosomal subunit protein uL14m	-11.259	0.681	-11.021	0.125	0.238	1.179	0.595	0.5839	0.85
Coq9	Q68FT1	Ubiquinone biosynthesis protein COQ9, mitochondrial	-11.273	0.752	-11.574	0.475	-0.301	0.812	-0.586	0.5894	0.8543
Slc25a40	Q498U3	Mitochondrial glutathione transporter SLC25A40	-12.698	0.15	-12.547	0.427	0.15	1.11	0.576	0.5957	0.8595
Acsf2	Q499N5	Medium-chain acyl-CoA ligase ACSF2, mitochondrial	-9.426	0.314	-9.265	0.4	0.162	1.119	0.551	0.6111	0.8779
Mrpl2	Q498T4	Large ribosomal subunit protein uL2m	-11.169	0.193	-11.243	0.138	-0.075	0.95	-0.545	0.6147	0.8794
Aldh2	P11884	Aldehyde dehydrogenase, mitochondrial	-6.21	0.184	-6.337	0.371	-0.127	0.916	-0.532	0.6228	0.882
Slc25a29	Q5HZE0	Mitochondrial basic amino acids transporter	-13.696	0.142	-13.79	0.274	-0.094	0.937	-0.529	0.6246	0.882
Mipep	Q01992	Mitochondrial intermediate peptidase	-12.392	0.647	-12.192	0.134	0.2	1.149	0.525	0.6274	0.882
Rhot2	Q7TSA0	Mitochondrial Rho GTPase 2	-11.388	0.286	-11.301	0.025	0.087	1.062	0.523	0.6285	0.882
Slc25a42	P0C546	Mitochondrial coenzyme A transporter SLC25A42	-11.635	0.744	-11.332	0.695	0.303	1.234	0.516	0.6332	0.882
Pdhb	P49432	Pyruvate dehydrogenase E1 component subunit beta, mitochondrial	-7.859	0.472	-8.034	0.372	-0.175	0.886	-0.505	0.64	0.882
Mt-nd4	P05508	NADH-ubiquinone oxidoreductase chain 4	-9.155	0.529	-9.314	0.151	-0.159	0.896	-0.5	0.6431	0.882
Mrps25	Q4QR80	Small ribosomal subunit protein mS25	-10.785	0.337	-10.899	0.216	-0.114	0.924	-0.494	0.6469	0.882
Atp5mpl	D3Z9R8	ATP synthase subunit ATP5MPL, mitochondrial	-9.208	1.038	-8.914	0.081	0.294	1.226	0.489	0.6505	0.882
Mtfr1	G3V9A7	Mitochondrial fission regulator 1	-13.942	1.255	-13.566	0.476	0.376	1.297	0.485	0.6532	0.882
Coq6	Q68FU7	Ubiquinone biosynthesis monooxygenase COQ6, mitochondrial	-5.593	0.182	-5.71	0.388	-0.117	0.922	-0.473	0.661	0.882
Timm44	O35094	Mitochondrial import inner membrane translocase subunit TIM44	-9.733	0.085	-9.772	0.116	-0.039	0.973	-0.47	0.6627	0.882
Supv3l1	Q5EBA1	ATP-dependent RNA helicase SUPV3L1, mitochondrial	-11.393	0.624	-11.579	0.28	-0.185	0.88	-0.469	0.6635	0.882

Gene	Accession	Protein	NOX_mean_log2	NOX_SD	HOX_mean_log2	HOX_SD	log2FC	FoldChange	t	p_value	FDR_BH
Atp5if1	Q03344	ATPase inhibitor, mitochondrial	-8.332	0.17	-8.512	0.645	-0.18	0.883	-0.468	0.664	0.882
Atp5f1c	P35435	ATP synthase F(1) complex subunit gamma, mitochondrial	-7.279	0.119	-7.217	0.203	0.062	1.044	0.459	0.6702	0.882
Mpc2	P38718	Mitochondrial pyruvate carrier 2;Isoform Short of Mitochondrial pyruvate carrier 2	-8.574	0.345	-8.319	0.9	0.255	1.193	0.458	0.671	0.882
Hmgcl	P97519	Hydroxymethylglutaryl-CoA lyase, mitochondrial	-9.782	0.271	-9.656	0.392	0.126	1.091	0.457	0.6712	0.882
Mrpl46	Q5RK00	Large ribosomal subunit protein mL46	-11.227	0.106	-11.343	0.426	-0.116	0.923	-0.457	0.6716	0.882
Aldh6a1	Q02253	Methylmalonate-semialdehyde/malonate-semialdehyde dehydrogenase [acylating], mitochondrial	-7.979	0.364	-8.105	0.317	-0.127	0.916	-0.455	0.6729	0.882
Txn2	P97615	Thioredoxin, mitochondrial	-12.335	0.838	-12.823	1.666	-0.488	0.713	-0.453	0.6739	0.882
Ndufs4	Q5XIF3	NADH dehydrogenase [ubiquinone] iron-sulfur protein 4, mitochondrial	-7.927	0.421	-8.051	0.248	-0.125	0.917	-0.443	0.6809	0.882
Atp5po	Q06647	ATP synthase peripheral stalk subunit OSCP, mitochondrial	-6.632	0.081	-6.602	0.082	0.029	1.02	0.439	0.6833	0.882
Tamm41	D3ZKT0	Phosphatidate cytidylyltransferase, mitochondrial	-11.718	0.239	-11.627	0.265	0.09	1.065	0.438	0.6837	0.882
Cox7c	P80432	Cytochrome c oxidase subunit 7C, mitochondrial	-9.219	0.466	-9.066	0.386	0.153	1.112	0.438	0.684	0.882
Mims1	Q5XIJ4	Mitochondrial inner membrane scaffold 1	-10.925	0.13	-10.882	0.121	0.043	1.03	0.422	0.6949	0.882
Acat1	P17764	Acetyl-CoA acetyltransferase, mitochondrial	-7.242	0.067	-7.201	0.157	0.041	1.029	0.42	0.696	0.882
Fastkd2	Q5M7V7	FAST kinase domain-containing protein 2, mitochondrial	-12.694	0.148	-12.659	0.023	0.036	1.025	0.412	0.7013	0.882
Cox7b	P80431	Cytochrome c oxidase subunit 7B, mitochondrial	-9.275	0.23	-9.439	0.652	-0.164	0.892	-0.412	0.7014	0.882
Fars2	Q6AYQ3	Phenylalanine--tRNA ligase, mitochondrial	-14.181	0.219	-14.272	0.318	-0.092	0.938	-0.412	0.7016	0.882
Timm13	P62076	Mitochondrial import inner membrane translocase subunit Tim13	-12.821	0.376	-12.708	0.298	0.114	1.082	0.41	0.7028	0.882
Tomm34	Q3KRD5	Mitochondrial import receptor subunit TOM34	-12.119	0.403	-11.988	0.381	0.131	1.095	0.408	0.7041	0.882
Oxct1	B2GV06	Succinyl-CoA:3-ketoacid coenzyme A transferase 1, mitochondrial	-6.944	0.164	-6.823	0.49	0.121	1.088	0.407	0.7048	0.882
Bcat2	O35854	Branched-chain-amino-acid aminotransferase, mitochondrial	-8.584	0.334	-8.684	0.264	-0.1	0.933	-0.407	0.7051	0.882
Micu3	A0A8I6A2H6	Calcium uptake protein 3, mitochondrial	-14.011	0.693	-14.173	0.087	-0.162	0.894	-0.401	0.7087	0.8831
Dld	Q6P6R2	Dihydrolipoyl dehydrogenase, mitochondrial	-7.123	0.159	-7.204	0.323	-0.081	0.945	-0.391	0.7155	0.8863
Acadsb	P70584	Short/branched chain specific acyl-CoA dehydrogenase, mitochondrial	-9.87	0.121	-9.804	0.271	0.067	1.047	0.39	0.7166	0.8863
Coq5	Q4G064	2-methoxy-6-polyprenyl-1,4-benzoquinol methylase, mitochondrial	-13.269	0.133	-13.32	0.191	-0.051	0.965	-0.378	0.725	0.8898
C1qbp	O35796	Complement component 1 Q subcomponent-binding protein, mitochondrial	-8.502	0.5	-8.639	0.402	-0.138	0.909	-0.371	0.7292	0.8898
Mt-co1	P05503	Cytochrome c oxidase subunit 1	-9.776	0.455	-9.952	0.691	-0.176	0.885	-0.369	0.731	0.8898
Tmem126b	B2RZD2	Complex I assembly factor TMEM126B, mitochondrial	-14.401	1.24	-14.707	0.73	-0.306	0.809	-0.368	0.7314	0.8898
Mff	Q4KM98	Mitochondrial fission factor	-12.721	0.341	-12.609	0.411	0.112	1.081	0.363	0.7351	0.8898
Vars2	Q6MG21	Valine--tRNA ligase, mitochondrial	-14.242	0.28	-14.158	0.289	0.084	1.06	0.362	0.7356	0.8898
Timm22	Q9JKW1	Mitochondrial import inner membrane translocase subunit Tim22	-12.969	0.855	-12.757	0.621	0.212	1.158	0.347	0.7458	0.8988
Bckdha	P11960	2-oxoisovalerate dehydrogenase subunit alpha, mitochondrial (Fragment)	-8.937	0.28	-8.994	0.081	-0.057	0.961	-0.34	0.7509	0.9016
Yars2	Q5I0L3	Tyrosine--tRNA ligase, mitochondrial	-12.162	0.322	-12.256	0.376	-0.094	0.937	-0.33	0.7577	0.9051
Ndufb1	P0DN35	NADH dehydrogenase [ubiquinone] 1 beta subcomplex subunit 1	-12.307	0.157	-12.525	1.139	-0.218	0.86	-0.328	0.7593	0.9051
Parl	Q3B8P0	Presenilin-associated rhomboid-like protein, mitochondrial	-12.263	0.365	-12.19	0.148	0.072	1.052	0.319	0.7658	0.9096
Pmpca	P20069	Mitochondrial-processing peptidase subunit alpha	-10.787	0.392	-10.716	0.117	0.071	1.051	0.303	0.7772	0.9198
Mt-nd5	P11661	NADH-ubiquinone oxidoreductase chain 5	-8.24	0.045	-8.279	0.236	-0.039	0.973	-0.282	0.7921	0.9341
Clpb	Q9WTT2	Mitochondrial disaggregase	-11.131	0.188	-11.075	0.307	0.056	1.04	0.269	0.8009	0.9391
Timm23	O35093	Mitochondrial import inner membrane translocase subunit Tim23	-12.276	0.668	-12.396	0.417	-0.121	0.92	-0.265	0.8038	0.9391
Mt-atp8	P11608	ATP synthase F(0) complex subunit 8	-11.044	0.301	-11.108	0.303	-0.065	0.956	-0.262	0.806	0.9391
Cox6c2	P11951	Cytochrome c oxidase subunit 6C-2	-8.399	0.26	-8.518	0.752	-0.119	0.921	-0.26	0.8078	0.9391
Mrrf	Q5RKI9	Ribosome-recycling factor, mitochondrial	-11.929	0.127	-11.972	0.265	-0.043	0.971	-0.253	0.8125	0.9413

Gene	Accession	Protein	NOX_mean_log2	NOX_SD	HOX_mean_log2	HOX_SD	log2FC	FoldChange	t	p_value	FDR_BH
Mrpl3	P18665	Large ribosomal subunit protein uL3m	-10.418	0.604	-10.512	0.367	-0.094	0.937	-0.23	0.8297	0.9511
Mrps15	Q5XI37	Small ribosomal subunit protein uS15m	-11.478	0.215	-11.438	0.221	0.04	1.028	0.226	0.8324	0.9511
	P56571	ES1 protein homolog, mitochondrial	-8.194	0.035	-8.232	0.288	-0.038	0.974	-0.226	0.8325	0.9511
Ndufs6	P52504	NADH dehydrogenase [ubiquinone] iron-sulfur protein 6, mitochondrial	-9.475	0.813	-9.334	0.714	0.141	1.103	0.226	0.8326	0.9511
Auh	F1LU71	Methylglutaconyl-CoA hydratase, mitochondrial	-9.862	0.328	-9.904	0.058	-0.043	0.971	-0.221	0.8357	0.9513
Abhd18	Q4V7A8	Cardiolipin-specific deacylase, mitochondrial	-15.971	1.048	-15.835	0.35	0.136	1.099	0.213	0.8419	0.9552
Slc25a25	Q8K3P6	Mitochondrial adenyl nucleotide antiporter SLC25A25	-11.187	0.278	-11.224	0.169	-0.037	0.975	-0.199	0.8523	0.9601
Oma1	D3ZS74	Metalloendopeptidase OMA1, mitochondrial	-13.081	0.423	-13.027	0.206	0.054	1.038	0.197	0.8531	0.9601
Nln	P42676	Neurolysin, mitochondrial	-10.627	0.137	-10.603	0.166	0.024	1.017	0.195	0.855	0.9601
Slc25a46	Q5EB62	Mitochondrial outer membrane protein SLC25A46	-13.442	0.28	-13.48	0.233	-0.037	0.974	-0.177	0.8679	0.9712
Mlycd	Q920F5	Malonyl-CoA decarboxylase, mitochondrial;Isoform Cytoplasmic+peroxisomal of Malonyl-CoA decarboxylase, mitochondrial	-10.814	0.281	-10.85	0.25	-0.036	0.976	-0.164	0.8775	0.9756
Ndufa10	Q561S0	NADH dehydrogenase [ubiquinone] 1 alpha subcomplex subunit 10, mitochondrial	-8.237	0.115	-8.269	0.324	-0.032	0.978	-0.163	0.8786	0.9756
Msra	Q923M1	Mitochondrial peptide methionine sulfoxide reductase	-14.006	0.762	-14.077	0.129	-0.071	0.952	-0.16	0.8807	0.9756
Trmu	B1WC37	Mitochondrial tRNA-specific 2-thiouridylase 1	-12.03	0.067	-12.051	0.241	-0.022	0.985	-0.149	0.8886	0.976
Ndufaf6	D3ZN43	NADH dehydrogenase (ubiquinone) complex I, assembly factor 6	-13.836	0.27	-13.778	0.627	0.057	1.041	0.146	0.8913	0.976
Mpv17	Q5BK62	Mitochondrial inner membrane protein Mpv17	-11.823	0.49	-11.735	0.941	0.088	1.063	0.144	0.8925	0.976
Atp5mg	Q6PDU7	ATP synthase F(0) complex subunit g, mitochondrial	-9.481	0.919	-9.559	0.392	-0.079	0.947	-0.136	0.8983	0.976
Opa1	Q2TA68-3	Isoform 3 of Dynamin-like GTPase OPA1, mitochondrial	-9.229	0.166	-9.207	0.227	0.021	1.015	0.132	0.9013	0.976
Fh	P14408-2	Isoform Cytoplasmic of Fumarate hydratase, mitochondrial	-7.777	0.161	-7.763	0.082	0.014	1.01	0.131	0.9021	0.976
Idh2	P56574	Isocitrate dehydrogenase [NADP], mitochondrial	-6.601	0.084	-6.614	0.151	-0.013	0.991	-0.127	0.9048	0.976
Coq2	Q499N4	4-hydroxybenzoate polyprenyltransferase, mitochondrial	-13.258	0.632	-13.206	0.354	0.052	1.037	0.124	0.9075	0.976
Slc25a5	Q09073	ADP/ATP translocase 2	-5.524	0.053	-5.55	0.409	-0.026	0.982	-0.108	0.9192	0.976
Mrpl41	Q5BJX1	Large ribosomal subunit protein mL41	-11.016	0.707	-10.97	0.23	0.046	1.033	0.107	0.9196	0.976
Sdhd	Q6PCT8	Succinate dehydrogenase [ubiquinone] cytochrome b small subunit, mitochondrial	-9.441	0.15	-9.456	0.201	-0.015	0.99	-0.102	0.924	0.976
Slc25a3	P16036	Solute carrier family 25 member 3	-4.782	0.051	-4.767	0.256	0.015	1.01	0.097	0.9278	0.976
Trub2	Q5XFW2	Pseudouridylate synthase TRUB2, mitochondrial	-15.592	1.775	-15.488	0.546	0.103	1.074	0.096	0.9278	0.976
Gfm2	Q5BJP6	Ribosome-releasing factor 2, mitochondrial	-13.122	0.318	-13.098	0.298	0.024	1.017	0.095	0.9287	0.976
Ptcd1	A2VD13	Pentatricopeptide repeat-containing protein 1, mitochondrial	-15.125	0.971	-15.18	0.308	-0.055	0.963	-0.094	0.9298	0.976
Echdc3	Q3MIE0	Enoyl-CoA hydratase domain-containing protein 3, mitochondrial	-11.812	0.369	-11.848	0.599	-0.036	0.975	-0.089	0.9335	0.976
Dlst	Q01205	Dihydrolipoyllysine-residue succinyltransferase component of 2-oxoglutarate dehydrogenase complex, mitochondrial	-7.306	0.673	-7.265	0.579	0.042	1.03	0.082	0.9387	0.976
Mterf4	Q4G078	Transcription termination factor 4, mitochondrial	-15.212	0.785	-15.253	0.482	-0.041	0.972	-0.077	0.942	0.976
Slc8b1	Q6AXS0	Mitochondrial sodium/calcium exchanger protein	-12.771	0.736	-12.729	0.607	0.042	1.03	0.077	0.9423	0.976
Mtrf1l	Q4V7E5	Peptide chain release factor 1-like, mitochondrial	-13.025	0.24	-13.048	0.466	-0.023	0.984	-0.076	0.9432	0.976
Fahd2a	B2RYW9	Oxaloacetate tautomerase Fahd2a, mitochondrial	-9.907	0.24	-9.889	0.363	0.018	1.013	0.073	0.9456	0.976
Osgepl1	Q4V7F3	tRNA N6-adenosine threonylcarbamoyltransferase, mitochondrial	-13.729	0.28	-13.713	0.26	0.016	1.011	0.072	0.9464	0.976
Atp5f1a	P15999	ATP synthase F(1) complex subunit alpha, mitochondrial	-4.236	0.132	-4.242	0.096	-0.005	0.996	-0.057	0.9571	0.9832
Tmlhe	Q91ZW6	Trimethyllysine dioxygenase, mitochondrial;Isoform 2 of Trimethyllysine dioxygenase, mitochondrial	-9.69	0.219	-9.681	0.15	0.008	1.006	0.054	0.9592	0.9832
Dmac2l	Q5XIM4	ATP synthase subunit s, mitochondrial	-12.513	0.108	-12.5	0.466	0.013	1.009	0.047	0.9649	0.9859
Fahd1	Q6AYQ8	Oxaloacetate tautomerase FAHD1, mitochondrial	-10.792	0.521	-10.805	0.276	-0.013	0.991	-0.039	0.9709	0.9889
Mrpl24	Q66H47	Large ribosomal subunit protein uL24m	-10.305	0.368	-10.308	0.028	-0.003	0.998	-0.016	0.9879	0.9962

Gene	Accession	Protein	NOX_mean_log2	NOX_SD	HOX_mean_log2	HOX_SD	log2FC	FoldChange	t	p_value	FDR_BH
Coq3	Q63159	Ubiquinone biosynthesis O-methyltransferase, mitochondrial	-13.548	0.916	-13.539	0.462	0.009	1.006	0.015	0.9884	0.9962
Acads	P15651	Short-chain specific acyl-CoA dehydrogenase, mitochondrial	-9.442	0.149	-9.444	0.277	-0.002	0.999	-0.009	0.9934	0.9962
Ndufv2	P19234	NADH dehydrogenase [ubiquinone] flavoprotein 2, mitochondrial	-8.045	0.196	-8.044	0.175	0.001	1.001	0.007	0.9944	0.9962
Mrpl40	P83565	Large ribosomal subunit protein mL40	-11.555	0.275	-11.554	0.227	0.001	1.001	0.006	0.9958	0.9962
Fis1	P84817	Mitochondrial fission 1 protein	-10.345	0.041	-10.346	0.372	-0.001	0.999	-0.005	0.9962	0.9962

E. Proteins with p < 0.05 (exploratory)

Proteins reaching p < 0.05 in the exploratory proteome-wide comparison. No protein survived FDR correction. Supplemental Figure S1 is the volcano plot of all mitochondrial proteins from Table D: log2 fold change against -log10 p.

Gene	Accession	Protein	NOX_mean_log2	NOX_SD	HOX_mean_log2	HOX_SD	log2FC	FoldChange	t	p_value	FDR_BH
Tfam	Q91ZW1	Transcription factor A, mitochondrial	-10.134	0.02	-9.845	0.045	0.289	1.222	10.193	0.0005218	0.09702
Coq8a	Q5BJQ0	Atypical kinase COQ8A, mitochondrial	-11.894	0.337	-14.038	0.213	-2.144	0.226	-9.324	0.0007364	0.09702
Cox4i2	Q91Y94	Cytochrome c oxidase subunit 4 isoform 2, mitochondrial	-13.583	0.17	-11.577	0.377	2.006	4.017	8.406	0.001096	0.09702
Lonp1	Q924S5	Lon protease homolog, mitochondrial	-7.558	0.002	-7.773	0.045	-0.214	0.862	-8.246	0.00118	0.09702
Stoml2	Q4FZT0	Stomatin-like protein 2, mitochondrial	-8.289	0.082	-7.75	0.093	0.539	1.453	7.53	0.001665	0.1096
Ckmt2	P09605	Creatine kinase S-type, mitochondrial	-7.894	0.597	-10.651	0.424	-2.757	0.148	-6.522	0.002854	0.1292
Pisd	D3ZAW2	Phosphatidylserine decarboxylase proenzyme, mitochondrial	-12.371	0.072	-12.086	0.028	0.285	1.219	6.384	0.003089	0.1292
Pmpcb	Q03346	Mitochondrial-processing peptidase subunit beta	-9.407	0.063	-9.776	0.082	-0.369	0.774	-6.161	0.003524	0.1292
Hspa9	P48721	Stress-70 protein, mitochondrial	-5.632	0.069	-6.049	0.1	-0.417	0.749	-5.949	0.004005	0.1292
Cyp27a1	P17178	Sterol 26-hydroxylase, mitochondrial	-12.368	0.084	-13.195	0.236	-0.827	0.564	-5.709	0.004655	0.1292
Lrpprc	Q5SGE0	Leucine-rich PPR motif-containing protein, mitochondrial	-9.001	0.075	-8.709	0.053	0.292	1.225	5.482	0.005393	0.1292
Acss3	A0A0G2K047	Acyl-CoA synthetase short-chain family member 3, mitochondrial	-14.083	0.22	-14.886	0.127	-0.803	0.573	-5.466	0.005449	0.1292
Mrpl11	Q5XIE3	Large ribosomal subunit protein uL11m	-11.761	0.214	-11.05	0.072	0.711	1.637	5.462	0.005464	0.1292
Atp5mf	D3ZAF6	ATP synthase F(0) complex subunit f, mitochondrial	-8.897	0.268	-7.784	0.239	1.113	2.163	5.37	0.005807	0.1292
Cox6a2	P10817	Cytochrome c oxidase subunit 6A2, mitochondrial (Fragment)	-11.368	0.1	-14.582	1.036	-3.214	0.108	-5.348	0.005892	0.1292
Slc25a15	A0A0G2K309	Mitochondrial ornithine transporter 1	-12.634	0.121	-13.014	0.037	-0.379	0.769	-5.178	0.006617	0.134
Glud1	P10860	Glutamate dehydrogenase 1, mitochondrial	-6.035	0.179	-5.422	0.105	0.613	1.53	5.112	0.006925	0.134
Gls	P13264-2	Isoform 2 of Glutaminase kidney isoform, mitochondrial	-9.011	0.073	-8.329	0.227	0.682	1.604	4.953	0.007742	0.1361
Pdp1	O88483	[Pyruvate dehydrogenase [acetyl-transferring]]-phosphatase 1, mitochondrial	-11.236	0.25	-10.389	0.166	0.847	1.799	4.88	0.008162	0.1361
Eci1	P23965	Enoyl-CoA delta isomerase 1, mitochondrial	-7.856	0.137	-7.332	0.13	0.524	1.438	4.798	0.008659	0.1361
Gpd2	P35571	Glycerol-3-phosphate dehydrogenase, mitochondrial	-11.126	0.033	-10.675	0.161	0.451	1.367	4.748	0.008987	0.1361
Suox	Q07116	Sulfite oxidase, mitochondrial	-12.595	0.17	-13.425	0.254	-0.829	0.563	-4.698	0.00932	0.1361
Higd1a	Q8VH49	HIG1 domain family member 1A, mitochondrial	-12.676	0.51	-11.231	0.194	1.445	2.722	4.589	0.01011	0.1361
Prdx5	Q9R063	Peroxiredoxin-5, mitochondrial;Isoform Cytoplasmic+peroxisomal of Peroxiredoxin-5, mitochondrial	-9.022	0.337	-8.124	0.038	0.898	1.863	4.587	0.01013	0.1361
Mecr	Q9Z311	Enoyl-[acyl-carrier-protein] reductase, mitochondrial	-11.585	0.233	-12.543	0.28	-0.957	0.515	-4.56	0.01034	0.1361
Isca1	Q80W96	Iron-sulfur cluster assembly 1 homolog, mitochondrial	-12.493	0.138	-12.057	0.104	0.436	1.353	4.376	0.01191	0.1503
Uqcrq	Q7TQ16	Cytochrome b-c1 complex subunit 8	-8.956	0.248	-8.244	0.146	0.712	1.638	4.284	0.01281	0.1503
Ndufv3	Q6PCU8	NADH dehydrogenase [ubiquinone] flavoprotein 3, mitochondrial	-10.723	0.249	-10.059	0.107	0.664	1.585	4.251	0.01315	0.1503
Ecsit	Q5XIC2	Evolutionarily conserved signaling intermediate in Toll pathway, mitochondrial	-11.175	0.065	-11.53	0.13	-0.356	0.782	-4.242	0.01325	0.1503
Acadvl	P45953	Very long-chain specific acyl-CoA dehydrogenase, mitochondrial	-8.525	0.121	-9.023	0.183	-0.498	0.708	-3.935	0.01703	0.1867
Fxn	D3ZYW7	Frataxin, mitochondrial	-13.142	0.047	-12.382	0.342	0.76	1.693	3.818	0.01881	0.1996
Idh3g	P41565	Isocitrate dehydrogenase [NAD] subunit gamma 1, mitochondrial	-8.219	0.086	-8.588	0.15	-0.369	0.774	-3.69	0.02102	0.2161
Gls	P13264	Glutaminase kidney isoform, mitochondrial	-6.856	0.327	-6.179	0.061	0.677	1.599	3.524	0.02436	0.2429
Clybl	Q5I0K3	Citramalyl-CoA lyase, mitochondrial	-12.73	0.114	-13.206	0.228	-0.477	0.719	-3.245	0.03152	0.305
Slc25a11	P97700	Mitochondrial 2-oxoglutarate/malate carrier protein	-8.126	0.229	-7.677	0.088	0.449	1.366	3.177	0.03365	0.3152
Letm1	Q5XIN6	Mitochondrial proton/calcium exchanger protein	-8.115	0.107	-7.837	0.11	0.277	1.212	3.131	0.03514	0.3152
Acadm	P08503	Medium-chain specific acyl-CoA dehydrogenase, mitochondrial	-8.224	0.143	-7.825	0.17	0.399	1.319	3.11	0.03586	0.3152

Gene	Accession	Protein	NOX_mean_log2	NOX_SD	HOX_mean_log2	HOX_SD	log2FC	FoldChange	t	p_value	FDR_BH
Bdh1	P29147	D-beta-hydroxybutyrate dehydrogenase, mitochondrial	-9.001	0.093	-8.226	0.427	0.775	1.711	3.073	0.03718	0.3152
Ca5b	Q66HG6	Carbonic anhydrase 5B, mitochondrial	-13.32	0.085	-14.869	0.87	-1.549	0.342	-3.068	0.03736	0.3152
Ivd	P12007	Isovaleryl-CoA dehydrogenase, mitochondrial	-8.221	0.305	-8.761	0.06	-0.539	0.688	-3.008	0.03962	0.3259
Top1mt	Q6IM78	DNA topoisomerase I, mitochondrial	-14.621	0.348	-13.87	0.284	0.751	1.683	2.894	0.04438	0.3562
Dhodh	Q63707	Dihydroorotate dehydrogenase (quinone), mitochondrial	-12.038	0.188	-11.359	0.364	0.679	1.601	2.866	0.04563	0.3574
Hspd1	P63039	60 kDa heat shock protein, mitochondrial	-4.829	0.148	-5.384	0.309	-0.555	0.681	-2.802	0.04871	0.3715
